# Journey of ZnO quantum dots from undoped to rare-earth and transition metal-doped and their applications

**DOI:** 10.1039/d0ra08670c

**Published:** 2021-01-12

**Authors:** Pushpendra Singh, Rajan Kumar Singh, Ranveer Kumar

**Affiliations:** Department of Physics, Dr Harisingh Gour Central University Sagar 470003 M. P. India ranveerssi@yahoo.com rajanphysicssgo@gmail.com +91 9425635731; Department of Chemical Engineering, National Taiwan University Taipei Taiwan ROC

## Abstract

Currently, developments in the field of quantum dots (QDs) have attracted researchers worldwide. A large variety of QDs have been discovered in the few years, which have excellent optoelectronic, antibacterial, magnetic, and other properties. However, ZnO is the single known material that can exist in the quantum state and can hold all the above properties. There is a lot of work going on in this field and we will be shorthanded if we do not accommodate this treasure at one place. This manuscript will prove to be a milestone in this noble cause. Having a tremendous potential, there is a developing enthusiasm toward the application of ZnO QDs in diverse areas. Sol–gel method being the simplest is the widely-favored synthetic method. Synthesis *via* this method is largely affected by a number of factors such as the reaction temperature, duration of the reaction, type of solvent, pH of the solution, and the precipitating agent. Doping enhances the optical, magnetic, anti-bacterial, anti-microbial, and other properties of ZnO QDs. However, doping elements reside mostly on the surface of the QDs. The presence of doping elements inside the core is still a major challenge for doping techniques. In this review article, we have focused on pure, rare-earth, and transition metal-doped ZnO QD properties, and the various synthetic processes and applications. Quantum confinement effect is present in nearly every aspect of the QDs. The effect of quantum confinement has also been summarized in this manuscript. Furthermore, the doping of rare earth elements and transition metal, synthetic methods for different organic molecule-capped ZnO QDs, mechanisms for reactive oxygen species (ROS) generation, drug delivery system for cancer treatment, and many more application are discussed in this paper.

## Abstract 2

Currently, developments in the field of quantum dots (QDs) have attracted researchers worldwide.

## Graphical abstract

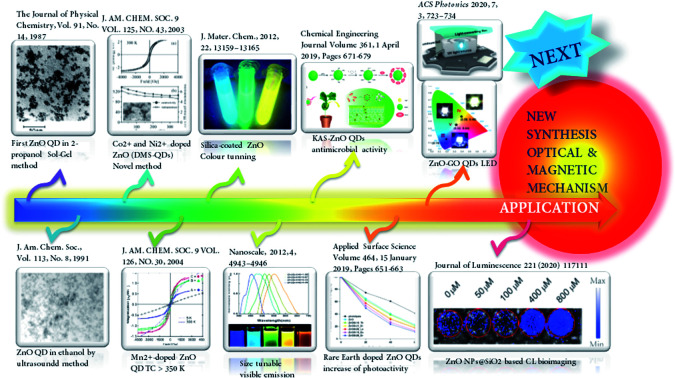

## 1. Introduction

From the past few years, efforts are being made for the development of stabilized colloidal systems. Different organic and inorganic (non-toxic) materials have been used to stabilize the colloidal systems, for example, Mesquite,^1^ milk fat globule membrane,^2^ Gum–chitosan mixture, xanthan gum, guar gum, and locust bean gum^3^ for the formation of multiple emulsions and proteins for the formation of foams.^4^ However, due to large industrial application of colloidal systems stabilized by solid particles, this is a hot area of research. Solid particles can be used in places where other stabilizers cannot be used, for example, at high temperatures. However, very little work has been done using solid particles as stabilizers.^5^ Some researchers have used solid particles to stabilize foam as these are easily adsorbed on the gas–liquid interface.^6,7^

Theoretically, QDs are crystals that have physical dimensions comparable to the exciton Bohr radius of the material of which they are made. These particles are confined in all the three dimensions. In case of ZnO QDs, it is found that the exciton Bohr radius is very small, which is nearly 0.9 nm. Thus, it is very difficult to synthesize ZnO QDs having radius less than the Bohr radius. Most of the ZnO QDs have a radius greater than the Bohr radius but comparable to it. These particles can be considered as quantum dots as they successfully show the effect of quantum confinement. It has an optical band gap of 3.37 eV, which further increases with the decrease in the particle size and large exciton binding energy (60 meV).^8^

**Fig. 1:**
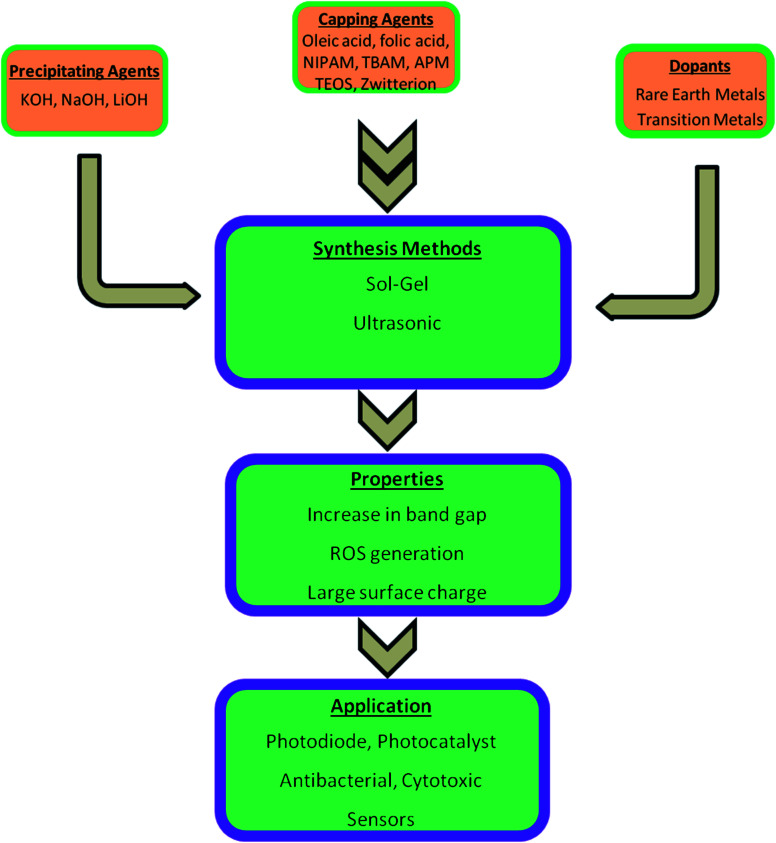
An overview of the synthesis of pure, doped, and conjugated ZnO, change in the properties, and their applications.

Most of the semiconductor quantum dots are in the form of colloidal solution. Among other colloidal systems, the colloidal solution of ZnO QDs is in great demand as they have a large area of application, excellent safety, good biocompatibility, non-toxicity, and low cost. Also, its anti-bacterial activity, biocompatibility, reliable mechanical properties, and physicochemical stability makes it a desirable candidate for dental materials. It is a potential candidate as a disinfectant and an antibacterial agent. These properties depend upon the structural morphologies and defects, surface functionalization, and exposure conditions. An illustrative diagram (Fig. 1) is provided for detailed information about the synthesis, properties, and applications of ZnO QDs. Hence, ZnO shows a wide range of applications in material engineering, health science,^9^ drug delivery^10,11^ photocatalyst, gas sensors,^12^ cancer therapy^13–15^ photoelectric light-emitting diodes (LED),^16–18^ solar cells,^19^ and photo-detectors.^20^

**Fig. 2:**
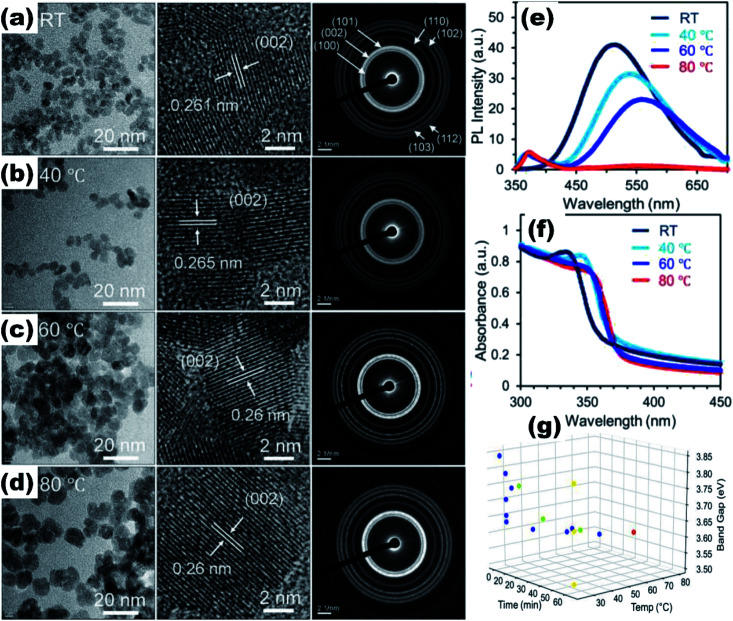
TEM, HRTEM, and SAED pattern of the ZnO QDs synthesized at (a) room temperature, (b) 40 °C, (c) 60 °C, and (d) 80 °C. (e and f) Photoluminescence and absorbance spectra of the ZnO QDs at different synthesis temperatures.^29^ (g) Training (blue) and testing (green, yellow and red) dataset of ZnO QDs. Adapted with permission from ref. 29. Copyright (2020) Elsevier.

Quantum confinement^21–23^ is a major aspect of QD that keeps it at the next level compared to nanoparticles. It has a direct influence over the optical properties of ZnO QDs.^24^ Three-dimensional quantum confinement of the charge carriers increases the life-time of the carriers and the photoluminescence intensity, which finally enhances the optoelectronic properties of the QDs. The quantum confinement effect can be easily understood as a “particle-in-a-box”. This phenomenon is observed when the electron wave-function is influenced by the size of the particles. For ZnO QDs having size less than 3.6 nm, a strong confinement is observed.^25^ To study the confinement effect practically, we need a 3D model. For such cases, “particle-in-a-box” is replaced by the “particle-in-a-sphere” model. In such cases, the expression for exciton binding energy can be written as1
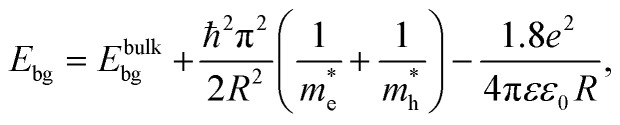
where *E*_bg_ is the band gap energy of the particle with radius *R*, *E*^bulk^_bg_ is the band gap energy for the bulk, 
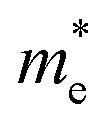
, 
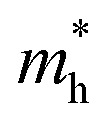
, *ε*, and *ε*_0_ are the effective mass of the electron, effective mass of the hole, dielectric constant for the semiconductor, and vacuum permittivity, respectively. The last term is the stabilization correction term for exciton binding energy.^26^ The theoretical study of exciton–phonon interaction and its effect on the ground state energy and oscillator strength shows a reduction with the particle size. R. T. Senger and K. K. Bajaj^27^ found that the polaronic self-energy corrections of the exciton vanishes completely and the PB potential effectually transforms into a dynamically-screened coulomb potential. In the PB model, the confinement effect on the exciton ground-state energy depends upon the exciton radius. The size of the exciton in very small dots depends on the boundary of the confinement potential. Quantum-confined Stark effect comes into existence with the decrease in the particle size (size less than 8 nm).^28^ In this effect, an electric field develops inside the particle due to the applied negative potential, which breaks the symmetry and splits the energy inside the conduction band. Experimentally, we can see the quantum confinement effect by studying the absorption and photoluminescence (PL) spectra (Fig. 2e and f) of the QDs. Both these spectra show a blue shift with a decrease in the particle size. From the TEM images, we can clearly see the decrease in the particle size (Fig. 2a–d). However, using ML, we can predict the different physical properties of the particles very accurately (Fig. 2g).^29^

**Fig. 3:**
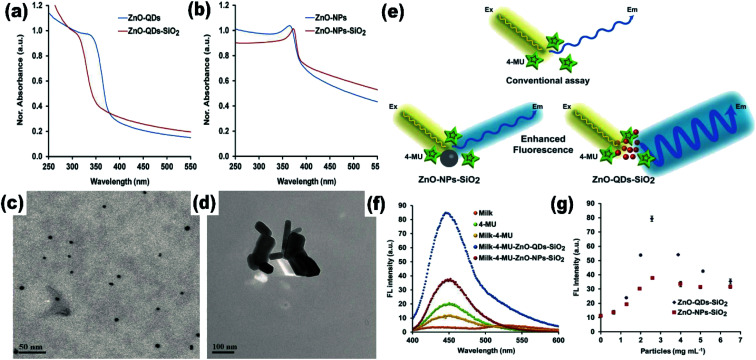
(a) and (b) UV-visible absorption spectra of SiO_2_-capped ZnO QDs and nanoparticles, respectively, (c and d) TEM images of SiO_2_-capped ZnO QDs and nanoparticles, (e) schematic illustration of the increase in the fluorescence spectra of a material by the QDs and the nanoparticles, (f and g) and variation in the fluorescence intensity.^41^ Adapted with permission from ref. 41. Copyright (2020) MDPI.

Quantum dots give excellent fluorescence as compared to nanoparticles due to the availability of the electron–hole pair to interact with the surface states of the quantum dots. The ZnO QDs significantly increase the fluorescence of the material as compared to the ZnO nanoparticles (Fig. 3e–g). ZnO QDs give yellow emission in the presence of UV light. The emission spectra may shift depending upon the precipitating agent used in the process, solvent,^30^ induced defects, and the size of the particles.^31^ The optical band gap of the QDs can also be tailored by the doping of different elements^32,33^ and by using capping agents. From Fig. 3a and b, we can see that by capping QDs with SiO_2_, the UV spectra gives a blue shift in the range from 340 nm to 310 nm, whereas on capping nanoparticles with SiO_2_, there is a red shift. This is attributed to the change in the particle size, which decreases upon capping in the QDs and increases in the nanoparticles (Fig. 3c and d). This is due to the restriction on agglomeration of ZnO QDs; the SiO_2_ coating plays the role of restricting the agglomeration. Liu *et al.*^34^ used Gd to reduce the size and increased the specific surface area of the ZnO QDs. Sun *et al.*^35^ found that besides the reduction in size and increase in the vacancy defects, La^3+^ also weakens the Zn–O bond, which give rise to more defects. However, excessive concentration of doping elements reduces the fluorescence emission intensity and the quantum yield.^36^ QDs also show the quenching of fluorescence intensity with the addition of the metal ion.^37^ The quenching of the fluorescence intensity can also be possible by the passivation of surface defects by the organic ligands. It was found that the intensity of the photoluminescence (PL) spectra of the doped QDs first increases with the concentration of the doping element and then starts decreasing with a further increase in the concentration of the doping elements. This is due to the weakening of the Zn–O bond with the increase in the doping concentration at the initial stage. This results in the production of more and more vacancy defects, which are responsible for the PL emission spectra. With a further increase in the concentration of the doping element, after a certain limit, the Zn–O bond breaks and the PL intensity starts quenching.^38,39^ Thus, ZnO QDs can be used as the fluorescence probe for the detection of metal ion impurities in drinking water as the detection limit for several ions is very low.^40^

ZnO QDs show strong anti-microbial properties at a specific pH^42^ as ZnO can be easily dissolved into Zn^2+^. Protected (QDs with any capping agent) QDs release Zn^2+^ ion at a specific location depending upon the capping agent.^43^ Thus, these are helpful in targeted drug delivery systems^44^ for the treatment of different diseases. Moreover, the concentration of the conduction band electron (e^−^) and valence band holes (h^+^) is very high even in the absence of UV (ultra-violet) light.^45^ The presence of more electrons and holes in the conduction and valence band, respectively, as compared to the QDs nanoparticles, shows more ROS (reactive oxygen species) generation. Hence, QDs have a large capacity to degrade organic molecules, microbes, and bacteria.^46^

**Fig. 4:**
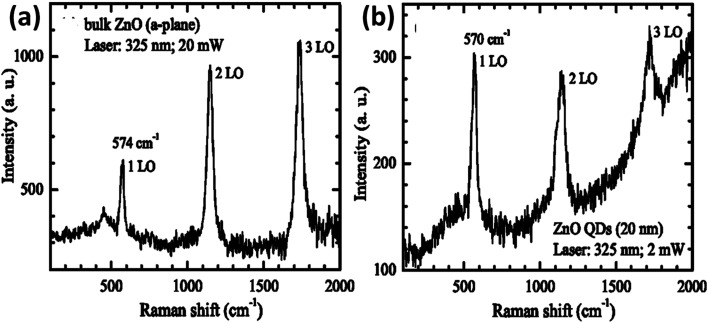
Resonant Raman spectra of (a) bulk ZnO nanoparticles and (b) ZnO QDs.^53^ Adapted with permission from ref. 53. Copyright (2005) AIP.

The effect of the particle size on the Raman spectra of ZnO was also found.^47^ In bulk ZnO, there are two phonon modes: longitudinal optical (LO) and transverse optical (TO).^48^ These modes further split into A_1_ and E_1_ symmetries. There are two non-polar Raman-active phonon modes with E_2_ symmetries also present. The low frequency E_2_ mode is related to the vibration of the zinc ion lattice and the high frequency E_2_ mode is related to the vibration of the oxygen ion lattice. The phonon peak shift that arises in the ZnO QDs is related to three main factors: phonon localization by defect creation, confinement effect within the QD boundaries, and laser-induced heating effect.^49–52^ Alim and his group^53^ studied the resonant and non-resonant Raman spectra of ZnO. They found that the E_2_ (high) peak shifted by 3 cm^−1^, which was due to the presence of defects. These defects are found to increase in the QDs and doped QDs in a large amount. They had also found that the resonant peak of the LO phonon mode shifts linearly towards the lower frequency side with the increase in the UV laser power. From Fig. 4a and b, we can see that there is a small shift of 4 cm^−1^ in the LO phonon mode for bulk ZnO to ZnO QDs. A change in the laser power shows a large red shift (in tens of cm^−1^) as compared to the other two factors (in few cm^−1^).

Since the past decade, there has been a surge in the field of ZnO QDs. This may be the outcome of congestion in the branch of nanoparticles, where researchers have not left any stone unturned. Yet we have not found any sizeable review article that can help to steer the research work forward. To overcome this drawback, we have tried to assemble all the material related to ZnO QDs in this review. In this review we have mainly focused on ZnO QDs. We have tried to cover most of the work done by researchers worldwide in this field in the last few decades. The main parts of this review are the synthetic approaches, doped ZnO QDs, and applications. We have added the effect of quantum size on the different physical properties of ZnO in the introduction part itself. After introduction, we discuss the synthetic approaches adopted by different researchers for the fabrication of ZnO QDs. This part covers the various ZnO QD fabrication methods, which includes the method for water and ethanol stable QDs. We have also tried to cover different synthetic methods, which include the capping of ZnO QDs so that the QDs remain stable for a large duration. We have also studied the synthetic methods for the doping of different elements. In the next section, we have studied the effect of doping in the QDs. Here, our main focus was on rare earth-doped QDs, transition metal-doped QDs, and composite QDs. We have also covered the effect of doping on various physical properties. The last section is the application part. Here, we have studied the fabrication of some devices in brief. We have also focused on some other application besides the devices. The main content of this section is bio-sensors, photodetectors, light emitting diodes (LED), catalytic application, as well as anti-cancer and anti-bacterial applications.

## 2. Synthetic approach

Among a number of techniques for the preparation of ZnO QDs (such as laser-induced fragmentation in liquid,^54^ radio-frequency (RF) atmospheric pressure plasmas (APPs)^55^) the sol–gel method is the most popular as it is relatively more efficient, simple, and inexpensive. This method was first adopted by Bahnemann^56^ for the preparation of ZnO QDs in the year 1987. They used 2-propanol as a solvent for the preparation of zinc acetate solution and NaOH was used for precipitation. The precipitate was finally suspended in water. Later, Spanhel^57^ used ethanol in place of 2-propanol for the synthesis of colloidal QDs. After that, in the last decade, many researchers have successfully synthesized QDs of ZnO using different solvents such as 2-propanol, ethanol, and water. Different hydroxides such as KOH, NaOH, and LiOH have been used as the precipitating agent. Solvents such as water, ethanol, and *n*-hexane have been used for the resuspension of the precipitate. Many research articles have emerged since then involving growth kinetics during the process, gelation, particle size control, use of capping agents, *etc.* Recently many researchers have successfully doped different elements in ZnO QDs.

### 2.1 Sol–gel synthesis

**Fig. 5:**
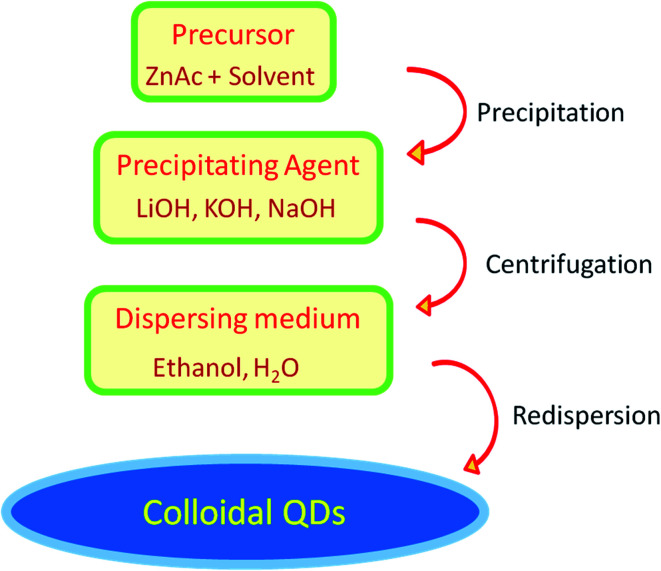
Schematic diagram for the synthesis of colloidal QDs of ZnO.

In this paragraph, we will discuss the typical sol–gel method for the synthesis of ZnO QDs. Mostly, zinc acetate (ZnAc) is used as a precursor material in this method. A schematic diagram of ZnO QD synthesis is shown in Fig. 5. The quantity of ZnAc is kept in the range of hundreds of micromoles to few a millimoles. Ethanol is mostly used to prepare the ZnAc solution. Temperature is kept in the range of 50° to 90 °C. A solution of the precipitating agent is also prepared simultaneously. For this solution, LiOH (lithium hydroxide), NaOH (sodium hydroxide), and KOH (potassium hydroxide) are also dissolved in ethanol. Finally, the solution of the precipitating agent is added in the ethanolic solution of ZnAc. A white precipitate is formed, which is then washed and collected by centrifugation. Generally, ethanol is used to wash the precipitate. The white precipitate is then resuspended in ethanol or water. The concentration of the zinc ion is kept low in the ethanolic solution; according to Ostwald theory,^58–60^ it will constrain the particle to grow to a larger size.

The reaction temperature and time also affect the particle size. Chen *et al.*^61^ studied the effect of reaction temperature and time. They used water–ethanol mixed solvent in the 2 : 1 ratio for the preparation of the ZnAc solution. Then, they mixed NaOH solution at 50, 60, 70, 80, and 90 °C for 6, 7, 8, 9, and 10 h reaction time. From both these variations, they have found that the particle size increases with the reaction temperature and time. The PL (photoluminescence) intensity of the QDs after 7 h of reaction was found to be the maximum. According to Chen,^61^ it was the time required to complete the reaction. Similarly, they found a higher emission peak for 60 °C reaction temperature. Regonia^29^ also studied the effect of reaction temperature and time on the optical properties of ZnO QDs. They found an increase in the particle size and decrease in the band gap. The increase in the particle size was confirmed by the TEM images of the QD grown at different temperatures. We can see that the particle size increases for synthesis at room temperatures, 40, 60, and 80 °C. The UV-visible absorption and photoluminescence spectra of the samples confirmed the decrease in the band gap energy. This variation can be easily defended by the Ostwald ripening theory as discussed earlier. They also studied the effect of different synthetic conditions on the physical properties of the QDs. Machine learning has proven to be the best supporting tool for predicting different physical properties. Instead of using artificial neural networks (ANN), they used Kernel ridge regression (KRR) and ridge regression (RR) as it needs very limited data sets for training the algorithm in comparison to ANN, which takes a large amount of data for training. In the field of nanomaterials, it is very tedious to take such a large amount of data. From Fig. 7 we can see that the KRR and RR model performs much better than the ANN model. The predicted band gap is very near to the experimental value.

**Fig. 6:**
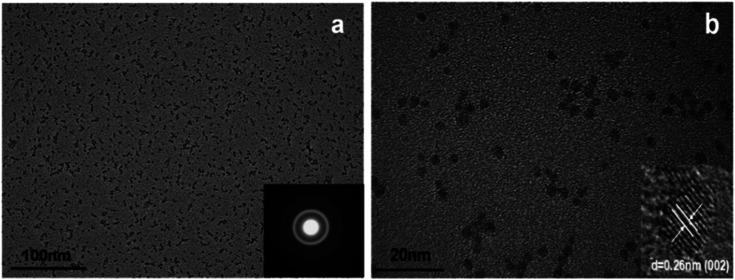
TEM images of ZnO QDs. (a) SAED pattern in the inset and (b) HRTEM in the inset.^62^ Adapted with permission from ref. 62. Copyright (2019) Elsevier.

A modified sol–gel method known as E. Meulenkamp's method can be adopted for the synthesis of ZnO QDs. This produces uniform and mono-disperse ZnO QDs by the precise control of the water content. In this method, first, a precursor solution of zinc salt (mostly zinc acetate) was formed in dehydrated ethanol and then to produce QDs from it, a calculated amount of water was added. Chen and his group^62^ studied the effect of the water content on the growth kinetics of ZnO QDs. The focus of these studies was on the two main processes, *i.e.*, oriented attachment (OA) and Ostwald ripening (OR). These result show that the water content affects both the processes. The TEM images of the QDs formed by this method are shown in Fig. 6. Fig. 6a shows the mono-disperse and uniform particles, and the SAED pattern in the inset. The crystal fringes shown in the inset of Fig. 6b confirm the 002 plane of ZnO (Table 1).

**Fig. 7:**
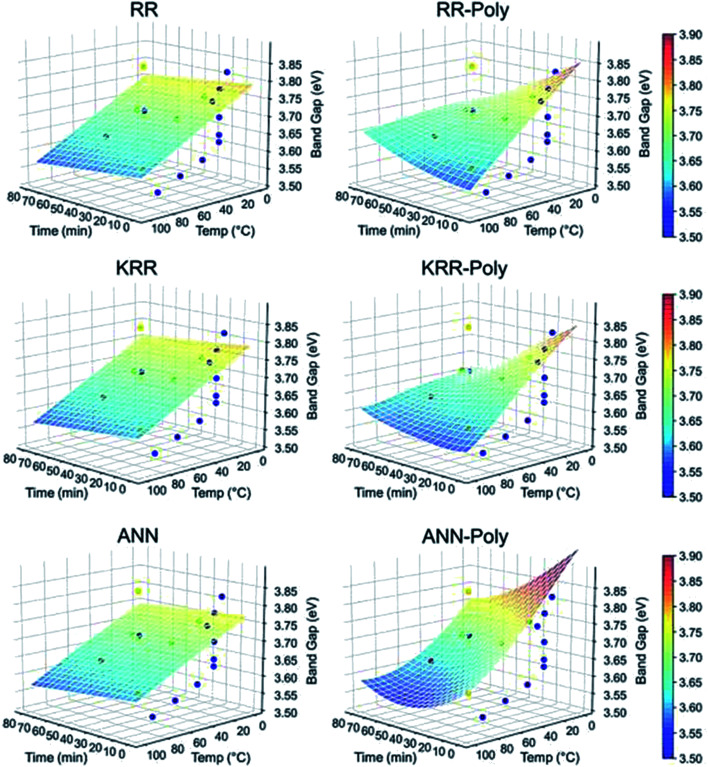
ML models for the band gap of ZnO QD at different temperatures and times.^29^ Adapted with permission from ref. 29. Copyright (2020) Elsevier.

**Table 1:** List of synthetic methods of different ZnO QDs and their properties

S. no.	Synthetic method	Solvent	Dopant	Capping agent	Composite materials	Change in properties	Ref.
1	Wet chemical	Ethanol	Co, Mn	—	—	Size 2.34 to 3.46 nm	63
2	Reflux at 80 °C for 2 h	Ethanol	La, Co	KH-560	—	The QY at 495 nm increases from 30.5% to 77.9%	35 and 64
3	Sol–gel	Ethanol	—	PEG	—	Selectivity toward the Cu^2+^ ion	65
4	Facile low-temperature solution process	Ethanol	—	PVP	—	Decomposes methyl orange	66
5	Solution method	Methanol and dispersed in water	—	TEOS	—	4–8 nm size, blue shift in PL emission	67
6	Ultrasonication microreactor	Ethanol	—	PEG-400	—	2–3 nm size, green emission, quantum yield 64.7%	68 and 69
7	One pot method	Ethanol	—	NIPAM, TBAM, APM		Labelling of *E. coli*, water stable for 15 days	70
8	Solution-based method	Ethanol	—	MSA	—	∼3 nm size, white light emitting	71
9	Wet chemical method	Ethanol	—	—	—	3–11 nm size	72
10	Sol–gel	Ethanol	—	—	—	6–10 nm size	73
11	Pulsed laser deposition (PLD) method and rapid thermal annealing	—	—	—	—	Average size 10 nm	74
12	Sonication	Ethanol	—	—	PMMA	Stable and flexible	75
13	Sol–gel	Ethanol	—	TEOS	—	White light emission	76
14	Modified sol–gel	Ethanol as the solvent and water for the QDs from the precursor solution	—	—	—	Water controls the QDs growth	62
15	Solution based method	Ethanol	—	KAS, EDC, APTS	—	pH triggered antimicrobial activity	77
16	Sol–gel	Ethanol	—	CTAB, TEOS	—	∼5 nm size, stable in saline water	78
17	Sol–gel	Ethanol	—	SBS	—	Stable under saline water for more than 30 days	79
18	Refluxed method	Ethanol	—	—	—	Cytotoxicity towards the MCF-7 and MDA-MB-231 cells	80
19	Sol–gel	Ethanol	—	Oleic acid and TMAH	PETTA and 2EEEA	Photooxidative degradation at selective sites	81
20	Hydrothermal	Ethanol–water mixed solvent	—	—	CuO sheet	Photocatalytic and antibacterial activity	82
21	Sol–gel	Ethanol	Pr^3+^	—	—	Energy transfers from the Pr^3+^ ions to the ZnO QDs.	32
22	Sol–gel	Ethanol	Mn^2+^	TOPO	—	Ferromagnetism	83
23	Sol–gel	Ethanol	Rare earth element	—	—	Photocatalytic and photoluminescence	84
24	Sol–gel	Ethanol	Cu	—	—	Increases field emission by nanorods	39
25	Ultrasonic method	Ethanol	tin	—	—	Changes direct band gap to indirect band gap	85

### 2.2 Coated ZnO QDs

Luminescent centers of ZnO QDs obtained by the classical sol–gel route are rapidly destroyed by water. For the biomedical application of ZnO QDs, it is important that QDs are stable in water. Several strategies have been deployed by researchers worldwide to obtain water-stable QDs by protecting the QDs with SiO_2_, oleic acid,^86^*etc.* It also prevents QDs from agglomeration.^87^ Zaiqian Yu^70^ and his group have used *N*-isopropylacrylamide (NIPAM), *N-tert*-butyl acrylamide (TBAM), and (*N*-(3-aminopropyl)methacrylamidehydrochloride) (APM) for the preparation of water-stable ZnO QDs.

**Fig. 8:**
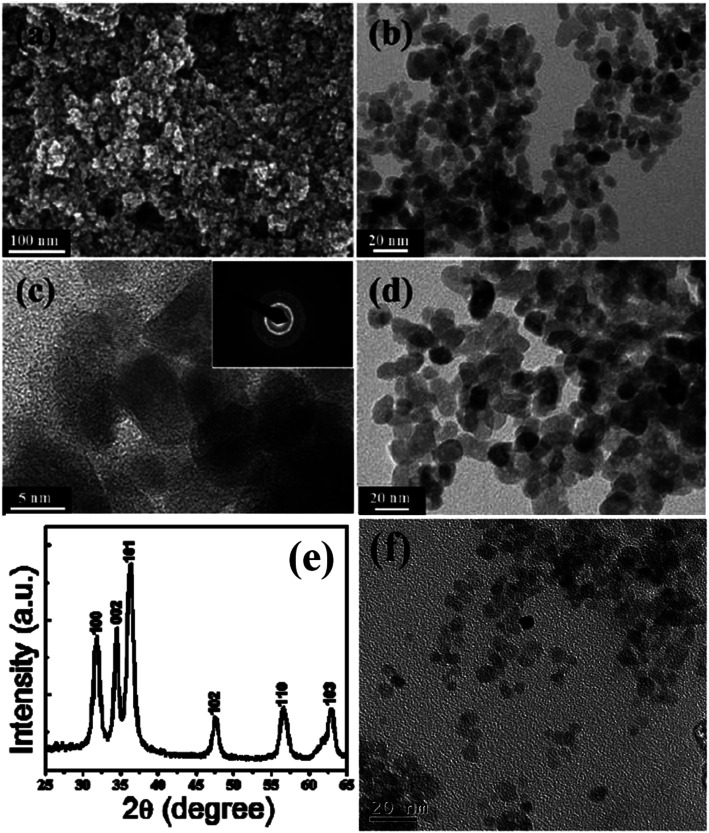
(a) FESEM image, (b) TEM image of small ZnO QDs, (c) high-resolution TEM image of the ZnO QDs; the corresponding SAED pattern in the inset, (d) TEM image of the large ZnO QDs, (e) XRD pattern of the ZnO QDs.^66^ Adapted with permission from ref. 66. Copyright (2014) Elsevier and (f) TEM image of the PEG-capped ZnO QDs.^65^ Adapted with permission from ref. 65. Copyright (2017) Elsevier.

Geng *et al.*^65^ used polyethylene glycol (PEG) for the preparation of water-stable monodisperse PEG capped ZnO QDs (shown in Fig. 8f). First, they prepared ZnO QDs in ethanol using ethyl acetate as the precipitating agent. Then, they added 4 mL PEG in 4 mL ZnO QDs and churned it at room temperature for 60 min. Finally, after centrifugation and washing with ethanol and ultrapure water, the QDs were obtained by dispersing them in 10 mL ultrapure water. Similarly, Rizwan Khan^66^ and his group used poly(vinylpyrrolidone) (PVP) for the photocatalytic application of ZnO QDs (shown in Fig. 8a–e). They dissolved 0.3 g ZnAC in an ethanolic solution of PVP (0.05 g PVP in 50 mL ethanol). After 5 min stirring at 70 °C, they added 0.1 g NaOH for precipitation. This precipitate was then centrifuged at 3000 rpm for 5 min and washed with ethanol 3–4 times. Zwitterion-coated water stable ZnO QDs have also shown stability towards the dissolved salts. Zhang *et al.*^79^ have prepared zwitterion-coated ZnO QDs and investigated the stability of 2 mg mL^−1^ QDs over a period of 30 days. They used saturated sodium chloride solution for testing. They did not find any remarkable change in the absorbance for 30 days. The luminescence intensity decreased only 1% when the sample was kept at 4 °C but at 37 °C, the luminescence intensity decreased to 30% after 1 day and remained 13% after 30 days. Recently, an excellent application of SiO_2_-coated ZnO has been found for the fabrication of an ink-absorbing and UV-shielding film. This film is was made by the dispersion of SiO_2_-coated ZnO in the PVA matrix and then spin-coating this solution on to the PET substrate.

**Fig. 9:**
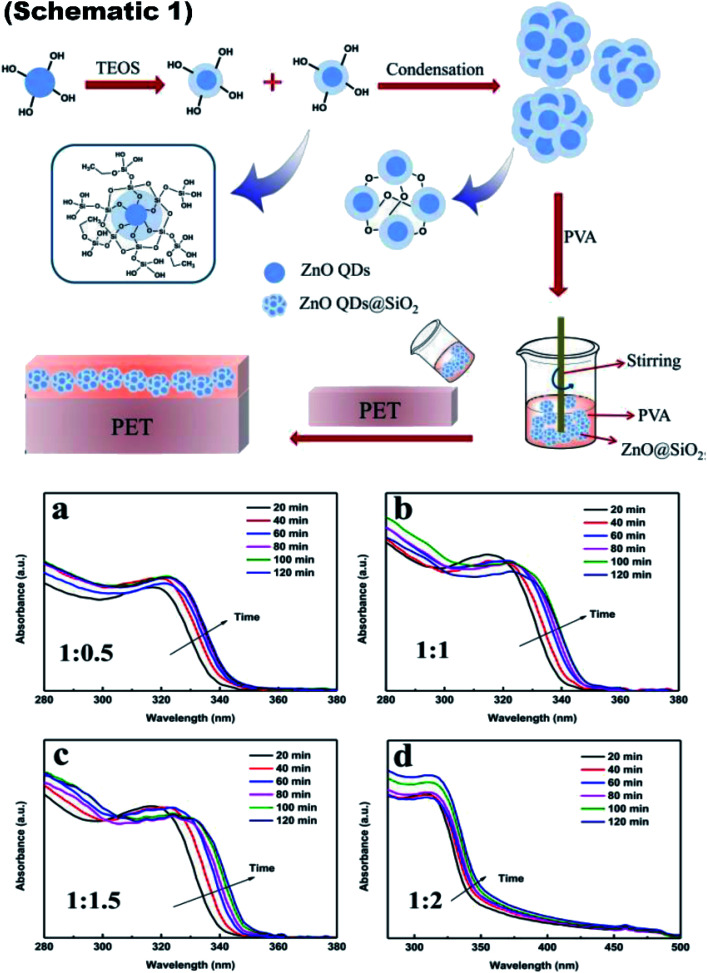
Schematic 1 Synthesis of ZnO QDs@SiO_2_ and its addition into PVA for the fabrication of the ink-absorbing coating, (a–d) UV-visible absorbance spectra of the ZnO QDs prepared with different ratios of Zn(Ac)_2_-to-NaOH at 1 : 0.5, 1 : 1, 1 : 1.5, and 1 : 2, respectively.^87^ Adapted with permission from ref. 87. Copyright (2020) Royal Society of Chemistry.

The schematic of the whole process is shown in Fig. 9, schematic 1. The UV-visible absorbance spectra is shown in Fig. 9a–d to show the UV absorbance property of the film. Graphs were made for the various ratio of zinc acetate and NaOH and various reaction time as both these factors directly affect the size of the ZnO QD. The size difference ultimately affects the band gap of the particle. From this, we can clearly see that as the ratio increases, the absorbance peak shifts towards the lower wavelength side, showing a decrease in the size of the ZnO QDs.^87^

### 2.3 Ultrasonic method

The ultrasonic method has been adopted by some researchers for the synthesis of stable ZnO QDs. In this method, ultrasonication is used for the preparation of different precursor solutions. In this method, the dissolution, oxidation, reduction, decomposition, and hydrolysis reactions take place in the liquid phase under ultrasonication.^69^

**Fig. 10:**
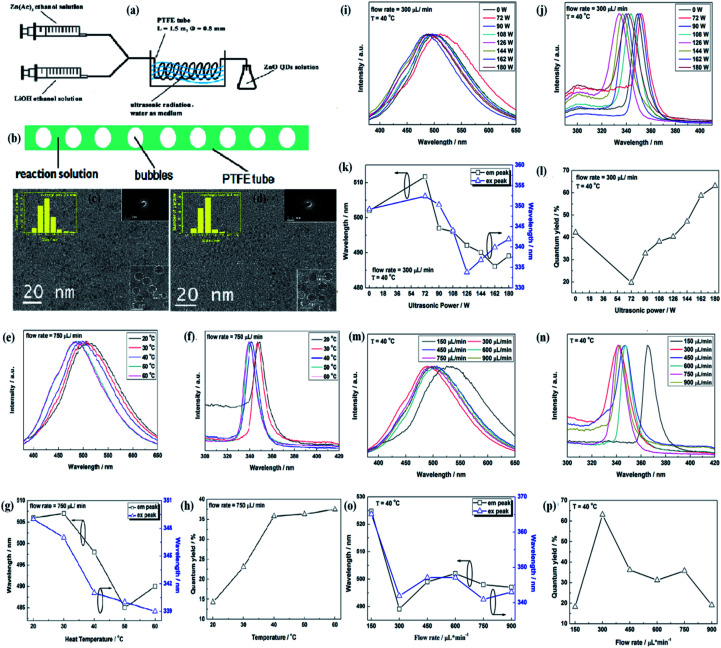
(a) Experimental setup of the ultrasonic microreactor, (b) simplified condition of the reaction solution in the tube under ultrasonication, (c and d) TEM and HRTEM micrographs, size distributions, electron diffraction patterns of the ZnO QDs synthesized under the flow rate of 300 lL min^−1^ and 750 lL min^−1^, respectively. The photoluminescence properties of ZnO QDs synthesized under the flow rate of 750 lL min^−1^ with 180 W ultrasonic power at different temperature. (e) Emission spectra, (f) excitation spectra, (g) emission and excitation peaks, and (h) quantum yield. Photoluminescence properties of the ZnO QDs synthesized at 40 °C under the flow rate of 300 lL min^−1^ with different ultrasonic power. (i) Emission spectra, (j) excitation spectra, (k) emission and excitation peaks, and (l) quantum yield. Photoluminescence properties of the ZnO QDs synthesized at 40 °C with 180 W ultrasonic power at different flow rates; (m) emission spectra, (n) excitation spectra, (o) emission and excitation peaks, and (p) quantum yield.^68^ Adapted with permission from ref. 68. Copyright (2016) Elsevier.

Weimin Yang and his group^68^ used the ultrasonication method and the micro-reactor method for the preparation of ZnO QDs; they named this method as the ultrasonic micro-reactor method (experimental setup is shown in Fig. 10a). In this method, two solutions, namely, zinc acetate and PEG-400 (*n*(PEG-400) : *n*(Zn) = 1 : 1) in 50 mL ethyl alcohol and LiOH in ethyl alcohol, were injected through separate syringes into a PTFE tube, which was immersed in an ultrasonic washer. Oleic acid was used to precipitate the QDs, which were then collected by centrifugation. In this process, ultrasonication creates bubbles in the reaction solution due to ultrasonic cavitation. These bubbles are shown in Fig. 10b. These bubbles divide the reaction solution into several parts and restrict the growth of the QDs on the surface of the bubbles as the tensile stress is more on the surface of the bubbles. The effect of the flow rate on the size of QDs is found to be negligible as both the QDs, prepared at 300 μL min^−1^ and 750 μL min^−1^ at 40 °C and 180 W power, have nearly similar average size (shown in Fig. 10c and d). They thoroughly studied the effect of ultrasonic power, flow rate, and temperature on the synthesis and optical properties of the QDs. Fig. 10g shows the effect of reaction temperature on the emission and excitation wavelength. The corresponding graph of photoluminescence emission and excitation are shown in Fig. 10e and f, respectively. These two graphs are in close relation with Fig. 10g. Fig. 10h shows the variation in the quantum yield with temperature. Fig. 10k shows the effect of ultrasonic power on the emission and excitation wavelengths. The corresponding graph of photoluminescence emission and excitation are shown in Fig. 10i and j, respectively. Fig. 10l shows the variation in the quantum yield with ultrasonic power. Fig. 10o shows the effect of flow rate on the emission and excitation wavelength. The corresponding graphs of photoluminescence emission and excitation are shown in Fig. 10m and n, respectively. Fig. 10p shows the variation in the quantum yield with the flow rate. They successfully obtained a quantum yield of nearly 42%.^68^

### 2.4 ZnO QD nanocomposite

**Fig. 11:**
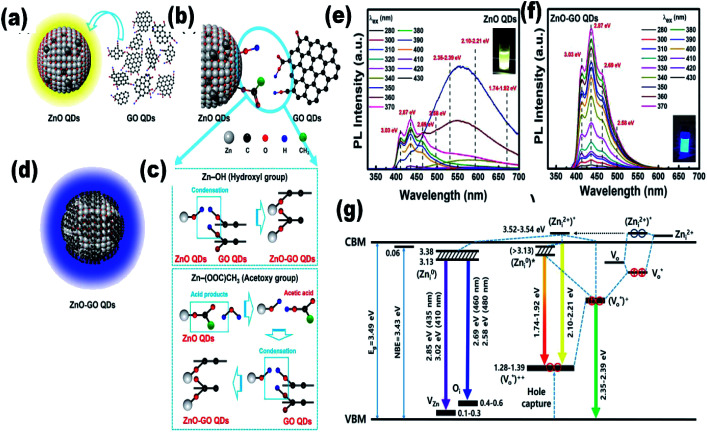
(a–d) Illustration of ZnO–GO formation, (e and f) PL spectra of the ZnO–GO composite and (g) the energy level of the ZnO–GO composite.^16^ Adapted with permission from ref. 16. Copyright (2020) American Chemical Society.

Since the last few years, researchers have been working on the development of flexible devices. To fabricate flexible devices, a polymeric substrate is the basic unit due to its liquid processing nature and flexibility for thin film fabrication. The fabrication of composite materials with ZnO QDs is beneficial for their long-term application. Graphene is an emerging candidate for QD composites. In recent years, graphene-based QD composites have attracted researchers worldwide.^88–90^ Nanocomposites made up of QDs and graphene have tremendous benefits over conventional materials. There are some features that make these materials superior over others, such as the strong quantum confinement effect in the QDs, exciton dissociation, high photoconductive gain, and charge transfer at the interface of the heterojunctions. The decoration of graphene by ZnO QD results in the enhancement of electron emission from graphene, which reduces the work function and ionization potential, and increases the Fermi level of graphene.^90^ The photo-responsivity and photoconductive gain of the ZnO QD/graphene heterojunction photodetectors can be enhanced to an extraordinary height if we are able to overcome the factors that hinder charge transfer at the interfaces. These factors are (1) contamination on the surface of the QDs and graphene; (2) layer of unreacted zinc acetate over ZnO QD, which creates a tunnel barrier; and (3) electron depletion layer due to adsorbed oxygen on the surface of the QD, which creates an interfacial barrier.^91^ It was found that a layer of reduced graphene oxide could turn paramagnetic ZnO QD ferromagnetic. It is possible only due to the hybridization of the 3d orbitals of the Co^2+^–oxygen vacancy complex and the 2p_*z*_ orbital of graphene.^89^ During the synthesis of the ZnO–GO composites, GO QDs first react with ZnO QDs. In this process, Zn–O–C and Zn–OOC hybrid bonding is formed by the reaction of hydroxyl group (Zn–OH) of ZnO QDs with the hydroxyl group (HO–C) and carboxylate group (HOOC) of GO QDs, respectively. The schematic synthesis diagram of these bonds is given in Fig. 11a–d. The PL spectra of the composite for different excitation wavelengths shows maximum intensity for wavelength *λ*_ex_ 350 nm having higher energy than the band gap energy (Fig. 11e). It is also interesting to note that the emission of violet–purple–blue emission is independent of the excitation wavelength (Fig. 11f). The energy level diagram in Fig. 11g is helpful in explaining the PL emission spectra of the composite.^16^

Some researchers have fabricated the ZnO–silica composite using tetraethoxysilane (TEOS). Recently, Liang *et al.*^76^ have mixed different amounts of ZnO QDs in the aqueous solution of TEOS. Patra and his group^67^ have also prepared ZnO QDs using TEOS. In a simple sol–gel method, first, they prepared an ethanolic solution of ZnAc. Then, they added KOH for maintaining the pH of the above solution at 10, 12, and 14. Then, they added TEOS to the above solution. The obtained colloidal solution was centrifuged and washed several times with methanol and water. Finally, the colloid was dispersed in water. Zain *et al.*^78^ also used TEOS for the synthesis of ZnO QD-embedded silica nanoparticles. They found 55% to 80% luminescence emission at 100 °C temperature and 40 g L^−1^ salinity. This increase in the stability was due to the hydrophobic properties of silica. You Liang and his group^77^ have prepared kasugamycin (KAS)-conjugated ZnO QDs for a pH-responsive pesticide delivery system. It was found to be more effective against bacterial fruit blotch as compared to pure KAS or ammonia-treated ZnO QDs.

**Fig. 12:**
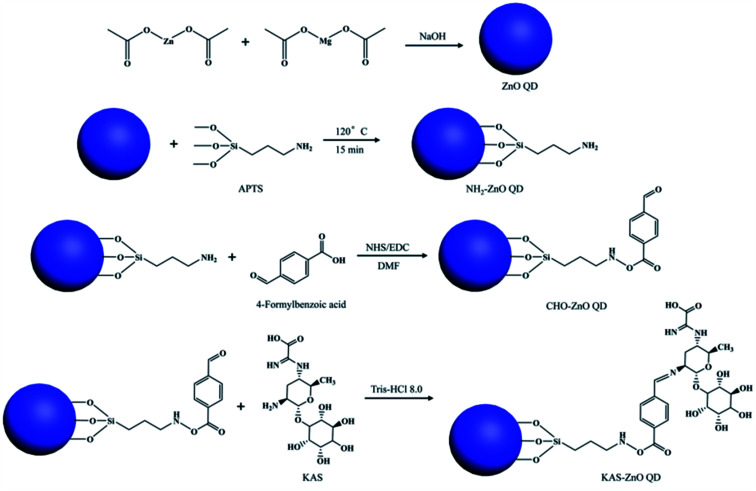
Synthesis mechanism for KAS-conjugated ZnO QDs.^77^ Adapted with permission from ref. 77. Copyright (2018) Elsevier.

For the synthesis of KAS-conjugated ZnO QDs, the QDs were prepared first, then (3-aminopropyl)trimethoxysilane (APTES) was modified on the surface of the QDs. The complete modification process is composed of three steps, which is shown in Fig. 12. From this figure, we can see that APTS-conjugated ZnO QDs then react with 4-formylbenzoic acid, followed by KAS to form the KAS-ZnO QDs.

### 2.5 Radio-frequency (RF)-based synthesis

Besides the above-mentioned solution routines, there are other different methods for the synthesis of ZnO QDs. Laser ablation, aerosol synthesis routes, chemical vapor synthesis (CVS),^92,93^ and radio-frequency (RF) plasma-based synthesis are some of them. These are environment-friendly and continuous flow processes with high production rate, and produce highly crystalline QDs. Radio frequency-based synthesis can be used for high purity samples. Vollath and his coworkers were the pioneers who had started the production of ZrO_2_, TiO_2_, and Al_2_O_3_. Later in 2002, Kleinwechter^94^ synthesized ZnO nanoparticles in the size range of 4 to 8 nm *via* aerosol synthesis routes using H_2_/O_2_/Ar plasma and O_2_/Ar plasma. In case of H_2_/O_2_/Ar plasma, the nucleation of ZnO nanoparticles starts outside the hot flame core, whereas Fe_2_O_3_ SiO_2_ forms inside the hot flame core. The particle size was in the range from 6.2 to 8.2 nm. In the case of O_2_/Ar microwave plasma synthesis method, the particles start nucleating in the plasma itself. The particle size was also found to be smaller than that of the above process; it was in the range of 4.2 to 5.9 nm. A change in the synthetic parameters not only affects the particle size but also affects different type of defects. It was found that with the increase in the hot wall reactor temperature, the stacking fault probability decreases for both (microwave plasma hot wall reactor and hot wall reactor) processes. However, the twin fault probability decreases only for the hot wall reactor process and increases slightly for the microwave plasma hot wall reactor process, whose twin fault probability is always lower than that of the hot wall reactor process.^96^ Later in 2013, Felbier^95^ and his coworkers synthesized ZnO QDs using radio-wave and non-thermal plasma instead of microwave. This process was largely scalable, ligand-free, and produces high quantum yield QDs. Recently Gunisha Jain^55^ has developed a method for the production of highly ligand-free, defect-free, hydroxyl-terminated ZnO QDs using radio frequency atmospheric pressure micro plasma. The particle size was also nearly 1.9 nm. The luminescence stability was also more than eighteen months. They used zinc wire instead of diethylzinc, which was the main precursor of zinc to date.

**Fig. 13:**
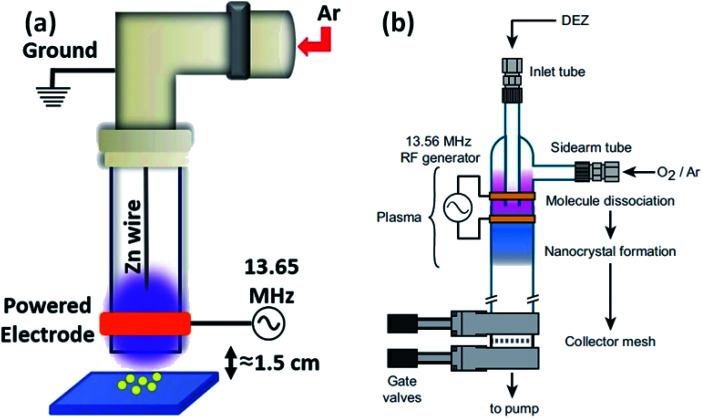
(a) Experimental set-up of the RF plasma reactor.^55^ Adapted with permission from ref. 55. Copyright (2020) IOP Science and (b) plasma reactor chamber.^95^ Adapted with permission from ref. 95. Copyright (2014) Wiley.

In summary, there is only one method based on the precipitation of zinc salt (mostly zinc acetate) in alcoholic medium. Different researchers have used different precipitating agents such as KOH, NaOH, and LiOH. A variation in precipitation results in a variation in the different physical properties. It was also found that at different pH values, we also get a variation in the band gap. Beside simple precipitating methods, there are some methods that are based on the ultrasonication of the precursor solution. Some methods have also been developed for more stable QDs, which are based on different capping agents such as SiO_2_, oleic acid, NIPAM, TBAM, APM, PEG, and PVP. The capping results are enthusiastic and produce more stable QDs as compared to pristine ZnO QDs. Conjugating organic compounds with QDs also results in stable QDs. Composites made in such a form are useful in a number of applications. The composites can be stable in salt water, which makes QDs applicable in saline conditions. Composites made up of graphene have tremendous potential in the near future (Fig. 13).

## 3. Doped ZnO QDs

Having unique properties in comparison to nanoparticles, QDs are very important in different application fields.^97^ The smaller size of the ZnO QDs not only widens the band gap of the QDs but also increases the surface to volume ratio. In addition to these surface defects, huge charge density is also created at the surface of the QDs. These unique properties of the QDs can be further modified with the addition of doping techniques. The doping of different elements gives direction to the above factors in various specific directions, which include photocatalytic, optical, magnetic, anti-microbial, and other properties. The doping of transition metal results in a thermally unstable material and generates electron–hole recombination centers.^98^

### 3.1 Rare earth metal-doped ZnO QDs

**Fig. 14:**
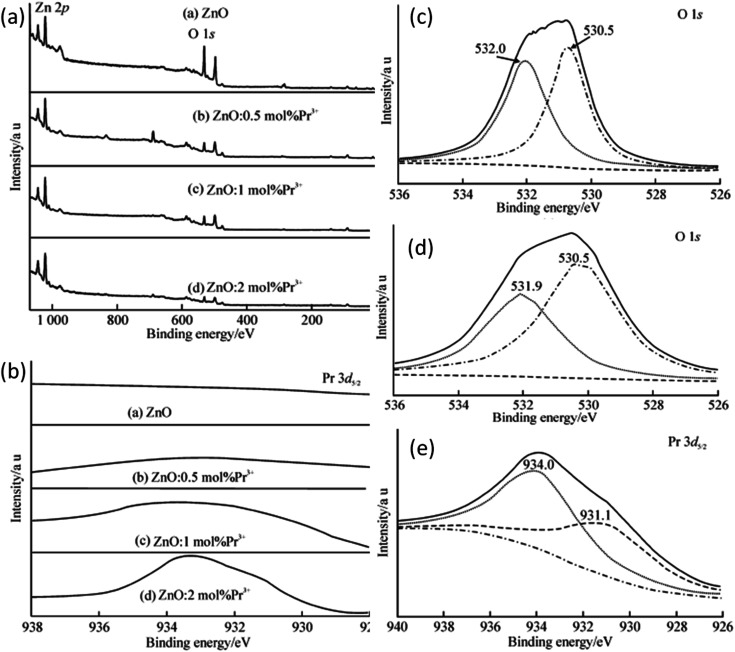
(a) XPS spectra of Pr^3+^-doped ZnO QDs, (b) binding energy scan of Pr^3+^-doped ZnO QDs for Pr 3d_5/2_, (c and d) fitting spectra of O 1s from undoped and 2 mol% Pr^3+^-doped ZnO QDs, respectively and (e) XPS spectrum of Pr 3d_5/2_ from 2 mol% Pr^3+^-doped ZnO QDs.^38^ Adapted with permission from ref. 38. Copyright (2014) Springer.

Rare earth elements, having more discrete energy levels, are very effective in photocatalytic and fluorescence applications of QDs. Hence, most of the researchers have focused on the study of rare earth-doped ZnO QDs. Due to the lesser number of atoms as compared to the nanoparticles, it is not easy to dope QDs. Hong *et al.*^38^ have found that most of the Pr^3+^ ions were on the surface of the QDs. The ions that penetrated into the particles caused lattice imperfection. The particle size was also found to decrease with the increase in the concentration of the doping element. With the increase in the Pr^3+^ concentration, the interaction between Pr^3+^ and the ZnO crystal hinders the growth of the crystal. This reduction can be explained by the interaction in between the Pr^3+^ ion and ZnO, which occurs on the surface of the crystal. Pr–Zn–O bonds form as a result of this interaction on the surfaces of the crystals. The XPS result confirms the formation of this bond. They thoroughly studied the XPS spectrum of pure, 0.5, 1, and 2 mol% Pr^3+^-doped ZnO QDs (shown in Fig. 14a) and found peaks at 531.5, 1044.9, and 1021.9 eV, which originate from O 1s, Zn 2p_1/2_, and Zn 2p_3/2_ photoelectrons, respectively. Besides these peaks, they also found an extra peak at 933.4 eV in Pr^3+^-doped ZnO QDs. This peak shows an extra 0.2 eV shift from the standard peak, which originates from the photoelectrons of Pr 3d_5/2_ in Pr_2_O_3_.

This result was due to the variation in the chemical bonding environment of Pr^3+^, which confirms the doping of Pr^3+^ in the ZnO lattice. From the XPS spectra of the Pr 3d_5/2_ peak (shown in Fig. 14e), we can observe an increase in the intensity with the increase in the doping concentration of Pr^3+^, which shows that the Pr^3+^ concentration increases in the ZnO QD with concentration. The spectrum of Pr 3d_5/2_ from 2 mol% Pr^3+^-doped ZnO QDs shows two peaks at 934.0 and 931.1 eV (shown in Fig. 14c). The higher binding energy component (HBEC) of Pr 3d_5/2_ at 934.0 eV is due to the formation of the Pr–O–Zn bond and the lower binding energy component (LBEC) of Pr 3d_5/2_ at 933.1 eV is due to the formation of the Pr–O–Zn bond in the electron rich-environment formed by oxygen vacancies. The O 1s spectra also have HBEC and LBEC similar to Pr 3d_5/2_ (shown in Fig. 14c and d). The HBEC of O 1s at 532.0 and 531.9 eV is due to the O–Zn bond surrounded by electron vacancies. The LBEC of O 1s at 530.5 eV is due to oxygen in the O–Zn bond. Both these spectra show a significant increase in the FWHM of all the components of LBEC and HBEC. This broadening is due to the increase in the distortion of the electron cloud of oxygen in the Pr^3+^-doped samples. As most of the doping elements are doped at the surface of the ZnO crystal and the XPS studies are also limited to a few nm of the crystal surface, it is an effective method for the confirmation of doping. From the XPS full spectrum scanning,^36^ we can see that the diffraction peaks of zinc and oxygen are only present before the doping of La. Sun *et al.*^35^ found that the size of ZnO QDs decreases with the increase in the La content.

**Fig. 15:**
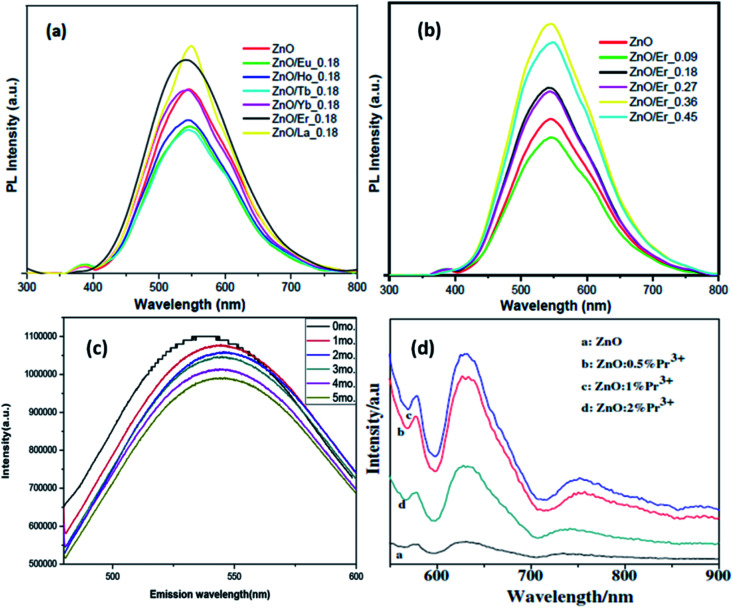
(a and b) PL spectra of different rare earth metal-doped ZnO QDs.^84^ Adapted with permission from ref. 84. Copyright (2018) Elsevier. (c) 5 mol% La-doped ZnO QDs at different intervals of time and (d) Pr^3+^-doped ZnO QDs.^32^ Adapted with permission from ref. 32. Copyright (2014) Elsevier.

Jakub Sowik^84^ and his group extensively studied the effect of different rare earth elements in different proportions on the optical, structural, and photocatalytic properties of ZnO QDs. The PL spectra of different rare earth metal-doped ZnO QDs is given in Fig. 15a and b. Here also, the XPS spectra of different rare earth elements show the binding energy shift in Zn 3d and the valence band spectra, which confirms the doping of rare earth elements. Er-Doped ZnO QDs was found to be the most effective towards the decomposition of phenol solution under visible light. The activity toward phenol decomposition was nearly 90% in UV. The PL intensity of La-doped ZnO QDs shows the highest PL quantum yield (nearly 80%). Gd doping is not only found to increase the quantum yield from 31% to 94% but also produces a red shift in the absorption and emission spectra.^99^ Similarly Huang *et al.*^36^ synthesized La-doped ZnO QDs and studied the effect of the OH^−^ ion and La concentration on the oxygen vacancy defect, which ultimately affects the fluorescence performance of the ZnO QDs. The oxygen vacancy was maximum for the 1 : 1 OH^−^ : Zn molar ratio and 7 mol% doping amount of La. The fluorescence emission intensity was found to be in direct proportion to the oxygen vacancy concentration. The fluorescence stability for 5 mol% doped QDs was nearly 5 months (shown in Fig. 15c). Rare earth doping will be very helpful in increasing the quantum efficiency of solar cells *via* down-conversion. Trivalent praseodymium could be an encouraging material as an activator for the host material. It was found that Pr^3+^doping effectively increases the PL intensity of some (578, 630, and 752 nm) defect-related emissions in the ZnO QDs.^32^ This increase in the emission is mainly related to energy transfer from the Pr^3+^ ion to the defect states.

From Fig. 15d, we can see that with the increase in the Pr concentration, the PL intensity also increases up to 1% doping concentration and then decreases for 2%. Thus, we can say that 1% is the doping limit for fluorescence applications. This decrease in the PL intensity could be a result of the concentration quenching effect. This effect is a result of cross relaxation and energy migration when the concentration of doping elements exceeds certain limits.

#### Gd doped ZnO QDs

**Fig. 16:**
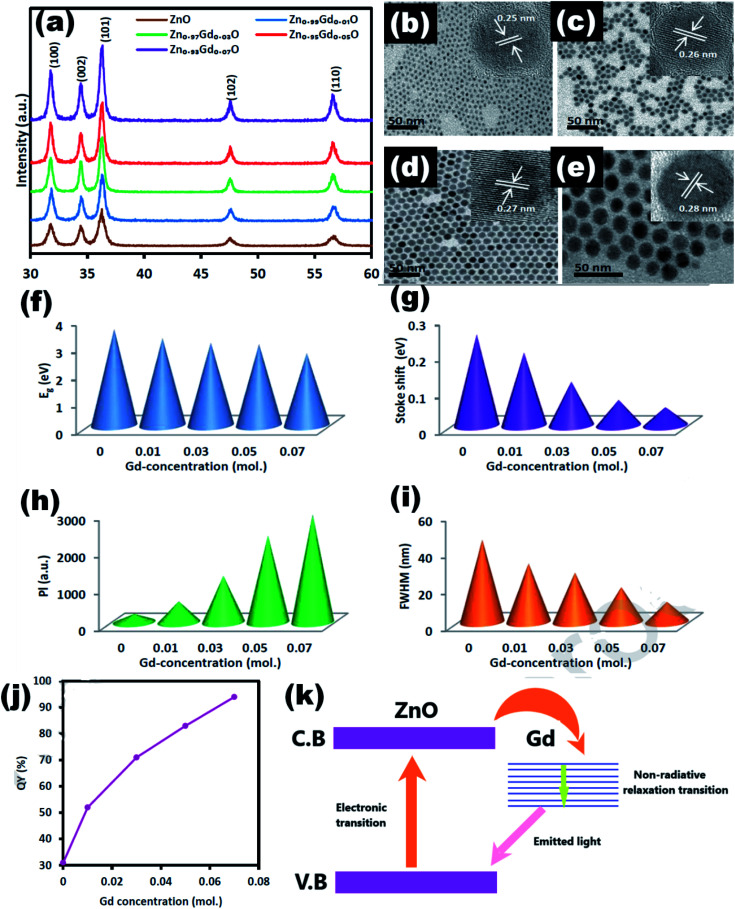
(a) XRD pattern of Zn_1−*x*_Gd_*x*_O, the TEM images of the Zn_1−*x*_Gd_*x*_O QDs at (b) *x* = 0, (c) *x* = 0.01, (d) *x* = 0.03, and (e) *x* = 0.07 (inset HRTEM images). The plot of (f) band gap, (g) Stokes shift, (h) PL intensity, and (i) FWHM in terms of the amount of Gd ions. (j) Quantum yield and (k) charge transfer mechanism.^99^ Adapted with permission from ref. 99. Copyright (2019) Elsevier.

Alshahrie *et al.*^99^ synthesized and studied Gd-doped ZnO QDs. It was found that Gd doping increases the crystallite size of the ZnO QDs while preventing their hexagonal structure. The XRD spectrum of Gd-doped ZnO QDs shows only a slight shift in the 101 peak, which may be due to the replacement of Zn by the Gd atom (shown in Fig. 16a). The TEM images show that the particle size increases from 3 to 18 nm with the increase in the concentration from 1 to 7% (Fig. 16b–e). The optical band gap and Stokes shift decrease with the concentration (Fig. 16f and g). The luminescence intensity increases six times due to the decrease in the crystal defects (Fig. 16h). However, FWHM decreases with the increase in the concentration (Fig. 16i). The quantum yield of luminescence also increases from 31 to 94% with doping (Fig. 16j). Fig. 16k shows the charge transfer mechanism in Gd-doped ZnO QDs. From this figure, we can see that Gd doping creates a band in the energy band gap of ZnO near the conduction band. Any excited electron goes to the conduction band of ZnO transfer to the conduction band of Gd *via* the relaxation non-radiative process. From this level, a very intense and coherent beam of light is produced.

### 3.2 Transition metal-doped ZnO QDs

Owing to the smaller size of transition metals, it is easier to dope them in ZnO QDs. There is more possibility of existence of doped transition elements inside the core as compared to rare earth elements. Radovanovic *et al.*^100^ fabricated Co-doped ZnO QDs by the iso-crystalline core/shell (ICS) procedure.^101^ As by the simple solution-based method, the doping elements reside mainly on the surface of the QDs. But by this method, his team successfully synthesized ZnO QDs with the doping elements in the core of the QDs. Transition metal-doping is also found to increase the photocatalytic and antibacterial properties of the ZnO QDs. Cadmium doping is found to be effective in increasing the production of ROS (reactive oxygen species), which plays a major role in the killing mechanism.^45^ Similar results were observed by Lee *et al.*^102^ for Al-doped ZnO QDs. The detection limit of mustard gas was 20 ppm. It was also found that these QDs show selectivity towards mustard gas.

Besides, rare earth transition metal-doped ZnO QDs have been found to show dilute magnetic properties.^103^ As a consequence of their tremendous application in spin-electronic and spin-photonic devices, vigorous investigation has to be done in the field of ferromagnetism in QDs. Magnetism in semiconductors arises due to the magnetic exchange interactions between the delocalized charge carriers and the localized magnetic impurities. These interactions result in large Zeeman and Faraday rotation effects. Mostly, in the early years, ligand-to-metal charge transfer(LMCT)^104,105^ transition was found to be the cause of ferromagnetism in doped ZnO QDs.^83^ In nickel and cobalt-doped ZnO QDs, the LMCT transition was also found to be responsible for ferromagnetism.^106^ This was also found to show a large Zeeman effect.^107,108^ In both these cases, there was a sub band gap in the energy level due to this LMCT. Beside this sub band gap, unique midgap excited states have been found by Joseph W. May in cobalt-doped ZnO QDs.^109^ Midgap transitions give rise to a number of properties such as magneto-electronic, magneto-optic, photocatalytic, and sensing properties. Stefan *et al.*^110^ later found a colloidal analogue to bind the magnetic polaron (BMP)^111^ responsible for magnetism in Mn^2+^-doped ZnO QDs. Yong^112^ and his group, *via* DFT theory,^85,113^ found that in the case of Mn^2+^-doped ZnO QDs, magnetism rises due to double exchange in the charge-transfer excited states. In case of p-type (N^2−^ doped) Mn-doped ZnO QDs, magnetic interaction between two Mn^2+^ ions is mediated by the N^2−^ ion. This results in parallel alignment of the spin of both the Mn^2+^ ion, which gives rise to ferromagnetism at room temperature.^114^

**Fig. 17:**
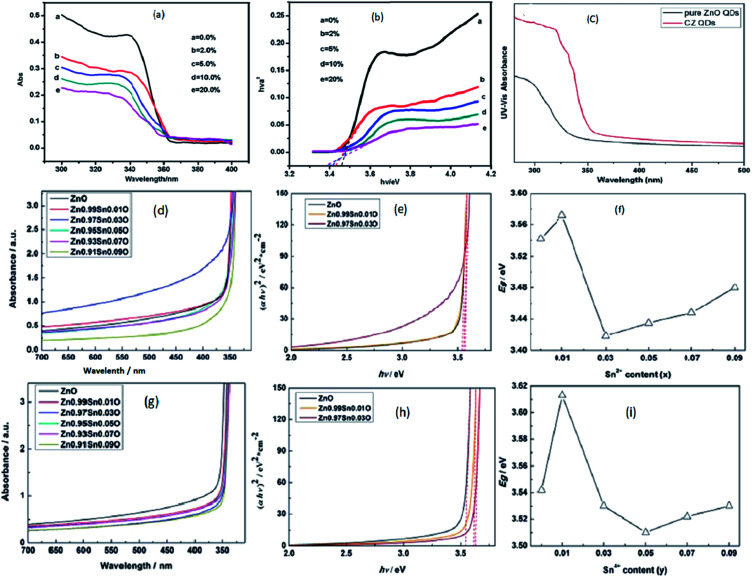
(a) UV-visible absorbance spectra of Cd-doped ZnO QDs at different concentration of Cd, (b) plots of (*αhν*)^2^*versus hν* of ZnO QDs with different concentrations of Cd.^33^ Adapted with permission from ref. 33. Copyright (2012) Springer. (c) UV-visible absorbance spectra of Cu-doped ZnO QDs.^39^ Adapted with permission from ref. 39. Copyright (2016) Elsevier. (d–f) UV-vis absorption spectra, plot of (*αhν*)^2^*vs.* (*hν*) and calculated *E*_gap_ of Sn^2+^-doped ZnO QDs, respectively. (g–i) UV-vis absorption spectra, plot of (*αhν*)^2^*vs.* (*hν*) and calculated *E*_gap_ of Sn^4+^-doped ZnO QDs, respectively.^85^ Adapted with permission from ref. 85. Copyright (2017) Royal Society of Chemistry.

Transition metal doping suppresses the growth of ZnO QDs and increases the various vacancy defects. Zhang *et al.*^33^ have also found similar results for Cd-doped ZnO QDs. With the increase in the Cd concentration, there was a blue shift in the UV spectra due to the quantum confinement effect. From Fig. 17a, a blue shift in the exciton absorption spectra was observed and from Fig. 17b, we can also see that there was a shift in the direct band gap for different concentrations of Cd. The PL intensity for the emission spectra was also found to increase with the increase in the doping concentration. Similarly, copper doping was also found to alter the energy band gap of the ZnO QDs. The energy band gap decreases with the concentration of Cu. From Fig. 17c, we can see that absorption increases with the doping concentration, which is mainly because of the substitution of Zn^2+^ by Cu^2+^. The substitution of Zn^2+^ results in an increase in the oxygen vacancies and electron concentration.^39^

With Sn doping, it was found that when the doping concentration of Sn was less than 3%, the doping was interstitial, and when the doping concentration was greater than 5%, the doping was substitutional.^85^ The UV-visible spectrum of Sn^2+^-doped ZnO QDs is shown in Fig. 9d and the corresponding change in the energy band gap is shown in Fig. 17e and f. From Fig. 17f, we can clearly see that with the increase in the Sn^2+^ concentration, initially, the band gap energy increases from 3.542 eV to 3.572 eV, then drops to 3.418 eV, and is finally increased to 3.480 eV. Similar results were shown by Sn^4+^-doped samples (shown in Fig. 17g–i). In case of the Sn^4+^-doped sample, the energy band gap first increased from 3.542 eV to 3.613 eV, then dropped to 3.510 eV, and finally rose to 3.530 eV. The *E*_gap_ reached the minimum value when the Sn^4+^ concentration was 0.05. This not only affects the binding energy of the O 1s spectrum but also affects the concentration of different types of defects present in the QDs.

#### Mg-Doped ZnO QDs

**Fig. 18:**
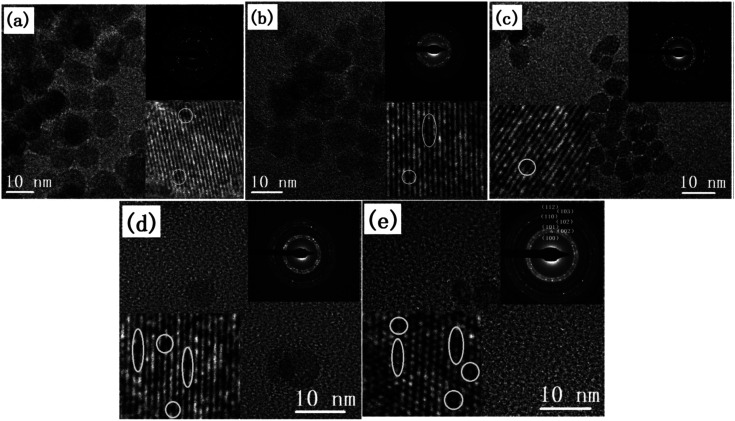
TEM images and SAED patterns of (a) undoped ZnO and Ce_*x*_Mg_0.1_Zn_0.9−*x*_O QDs with (b) *x* = 0, (c) *x* = 0.004, (d) *x* = 0.008, and (e) *x* = 0.01.^115^ Adapted with permission from ref. 115. Copyright (2020) Elsevier.

**Fig. 19:**
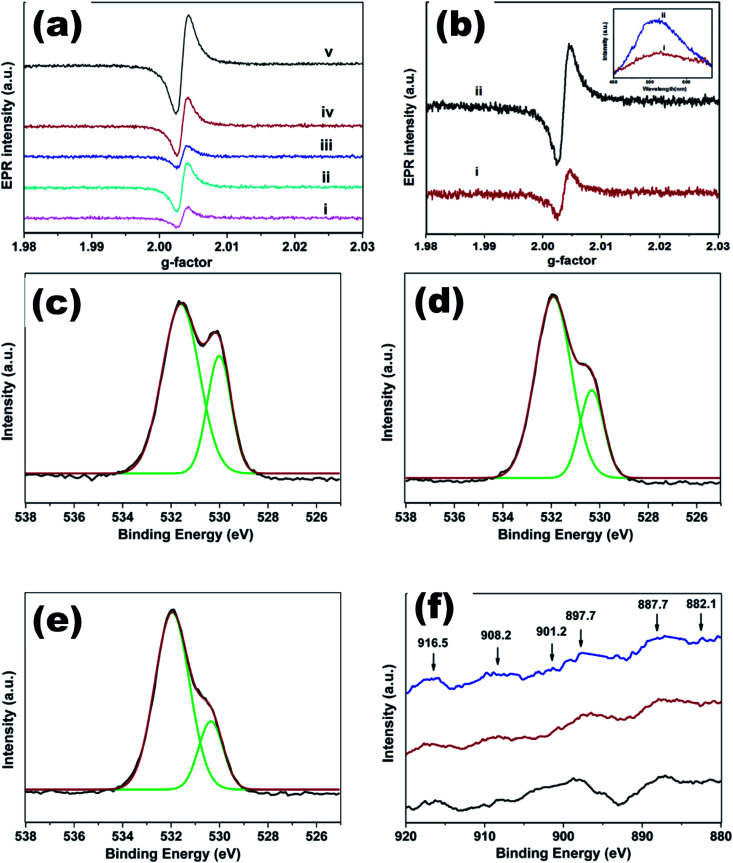
(a) EPR spectra of undoped ZnO and Ce_*x*_Mg_0.1_Zn_0.9−*x*_O QDs, (b) EPR and PL (inset) spectra of Ce_*x*_Mg_0.1_Zn_0.9−*x*_O QDs, O 1s XPS spectra of Ce_*x*_Mg_0.1_Zn_0.9−*x*_O QDs with (c) *x* = 0.004, (d) *x* = 0.008, (e) *x* = 0.01, and (f) Ce 3d XPS spectra of Ce_*x*_Mg_0.1_Zn_0.9−*x*_O QDs (*x* = 0.004, 0.008, 0.01).^115^ Adapted with permission from ref. 115. Copyright (2020) Elsevier.

Co-Doping in the ZnO QDs was first introduced by Shi *et al.*^115^ They fabricated Mg and Ce co-doped ZnO QDs *via* a simple facile low temperature route. The crystallite size was found to be decreasing with increasing Ce concentration. The TEM images also show that the particle size is decreasing with the increasing doping concentration (Fig. 18). The EPR spectra of all the samples shows a similar peak at *g* ≈ 2.0035, which is due to the presence of oxygen vacancies (shown in Fig. 19a and b). These signals become stronger after annealing the samples in a reducing atmosphere, which confirms that the EPR signals are from oxygen vacancies. The oxygen 1s XPS spectra (Fig. 19c–f) is composed of two peaks, the first at ∼530 eV and the other at ∼532 eV, corresponding to the lattice oxygen (O_L_) in ZnO and oxygen vacancies (O_H_) in the ZnO matrix, respectively. The ratio of O_L_/O_1s_ decreases from 31% to 21% with the increase in the Ce concentration and the ratio of O_H_/O_1s_ increases from 68.99% to 78.45% with the increase in the Ce concentration. This confirms that the oxygen vacancy increases with the Ce concentration, which is in accordance with the EPR spectra.

In summary, doping in QDs is not as easy as in nanoparticles. Doping mainly results in the smaller size of QDs as it restricts the particle growth. Doping also alters the energy band gap of the QDs, which causes a blue shift. Larger doping elements mostly reside on the surface of the QDs. But there are some methods that help the doping elements to reside at the core of the QDs. Rare earth doping mostly results in the manipulation of the luminescence properties. It is also possible to use these as photocatalysts. Besides, rare-earth doping and transition metal doping is found to show magnetism in the QDs. Here, doping elements not only reside on the surface but also inside the core up to some limit. Magnetic interactions between the spins are mostly LMCT and double exchange interactions. These result in room temperature ferromagnetism as well as large Zeeman and Faraday rotation effects.

## 4. Application

ZnO is a wide band gap semiconductor having a direct band gap of 3.37 eV. This band gap can further increase with the decrease in the particle size, which is characteristic of ZnO QDs. Besides, the modulation in the band gap QDs also differs in a number of ways such as modified charge carrier density,^116^ large surface-to-volume ratio, and the various energy levels that are distinct from those of nanoparticles. These mutations in the properties of the QDs are useful in some of the pioneer applications of the ZnO QDs, which are not found in nanoparticles. Impurity element detection is one of these applications, which is not applicable using nanoparticles. Apart from impurity detection, energy storage,^117^ gas and chemical sensing,^118^ optoelectronics,^20,119^ anti-bacterial,^120^ cancer treatment,^121,122^ cellular imaging,^123,124^ electronics,^125^ and photocatalytic activity^126^ are the other major application fields of QDs. However, these QDs will definitely augment the performance of these devices, which have based on nanoparticles to date. The surge in the application of QDs in different fields during the last few decades proves its golden future.

### 4.1 Detection of metal ions

**Fig. 20:**
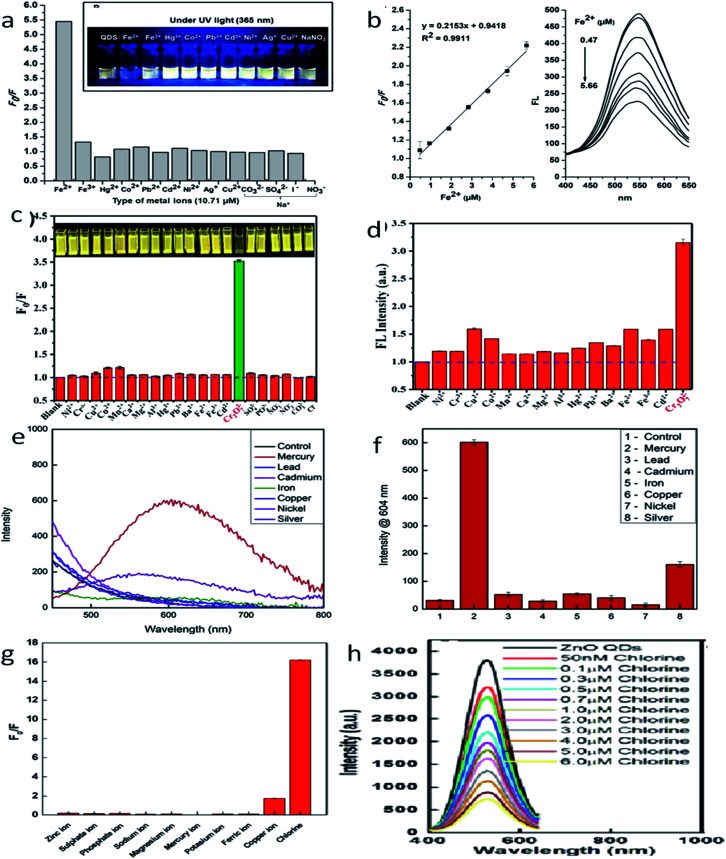
(a) *F*_0_/*F* for different ions, (b) fluorescence intensity of the Fe^2+^ ion at different concentrations.^40^ Adapted with permission from ref. 40. Copyright (2019) American Chemical Society. (c) *F*_0_/*F* for different ions, (d) fluorescence intensity of the Cr^6+^ ion at different concentrations.^37^ Adapted with permission from ref. 37. Copyright (2019) IOP Science. (e) Fluorescence intensity for different ions, (f) bar diagram for different ions.^146^ Adapted with permission from ref. 146. Copyright (2019) Elsevier. (g) *F*_0_/*F* for different ions and (h) fluorescence intensity of the chlorine ion at different concentrations.^147^ Adapted with permission from ref. 147. Copyright (2016) Royal Society of Chemistry.

The development of chemosensors for the detection of metal ion impurities^127^ in drinking water has become a hot area for intensive research. Many researchers are working in this field for the development of selective detection of transition and heavy metal ion impurities. From the different available detection methods such as colorimetry,^128–130^ mass spectrometry,^131–133^ atomic absorption spectroscopy,^134–136^ electrochemical^137–140^ and fluorescence^141–145^ methods, the fluorescence methods are the most reliable and effective because of their high sensitivity, high efficiency, and operative simplicity. Praethong Laopa and Tirayut Vilaivan^40^ synthesized cationic polymer-coated ZnO QDs for the selective detection of the Fe(ii) ion. The fluorescence study of p(METAC38-PEOMEMA62)–ZnO, p(METAC100–PEOMEMA0)–ZnO, and p(METAC9-PEOMEMA91)–ZnO QDs was investigated in the presence and absence of 10.71 μM metal ions such as Fe^2+^, Fe^3+^, Hg^2+^, Co^2+^, Pb^2+^, Cd^2+^, Ni^2+^, Ag^+^, Cu^2+^, and Na^+^. From Fig. 20a and b, we can see that the variation in the fluorescence intensity in the absence and presence of the ion (*F*_0_/*F*) for different ions is maximum for Fe^2+^, which shows selectivity towards the Fe^2+^ ion. We can also see that the fluorescence intensity for this ion decreases with the increase in the concentration of the ion. Similarly, Tong *et al.*^37^ used a mixture of urea and ZnO QDs for the detection of the Cr^6+^ ion. The detection limit of Cr^6+^ was 19.53 nM. Fig. 20c and d shows (*F*_0_/*F*) and the fluorescence intensity of different ions, respectively. This shows the selectivity towards the Cr^6+^ ion. Daniel^146^ and his team developed a hand-held device for the real time detection of the poisonous Hg^2+^ ion. The detection limit of the device is 0.1 ppb and it has a linear range of detection from 0.1 ppb to 10 000 ppb. From Fig. 20e, we can see the fluorescence spectra of ZnO QDs in the presence of different ions. The intensity for the Hg^2+^ ion is the highest, which shows the selectivity of the instrument towards this ion. The bar diagram shown in Fig. 20f confirms this result. The presence of chlorine in water can also be detected by the ZnO QDs.^147^

**Fig. 21:**
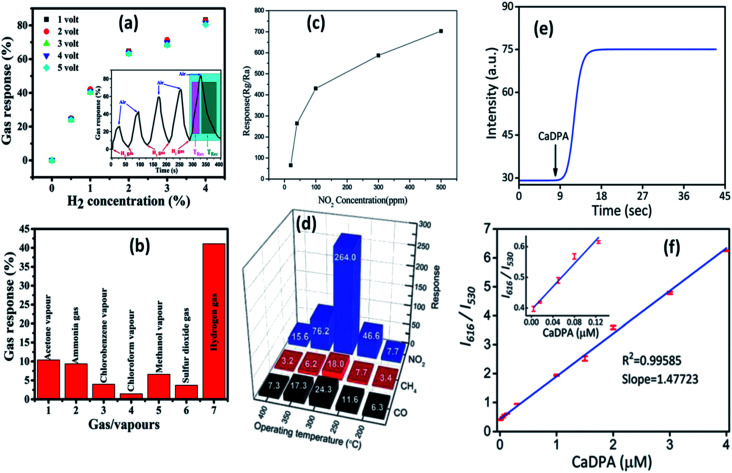
Gas response curve at (a) different operating voltages and H_2_ concentrations, the response and recovery curve in the inset, (b) different gases.^148^ Reprinted (adapted) with permission from ref. 148. Copyright (2019) IOP Science. Response curve for (c) different NO_2_ concentrations and (d) different gases.^149^ Adapted with permission from ref. 149. Copyright (2011) Royal Society of Chemistry. (e) Real time response curve and (f) ratiometric calibration curve for CaDPA detection.^151^ Adapted with permission from ref. 151. Copyright (2017) Royal Society of Chemistry.

As the concentration of chlorine increases, it absorbs electrons from the oxygen vacancies. This results in a decline in the emission intensity of the QDs. From Fig. 20g, we can see the *F*_0_/*F* for different ions, which shows selectivity towards chlorine in the solution. Fig. 20h shows the fluorescence spectra for the chlorine ion at different concentrations. Capping with different polymers or organic materials produces selectivity toward different polluting elements. These ZnO QD-based detecting solutions are very cheap and easy to handle. Thus, these QDs can become a useful tool for the detection of impurities (Fig. 21).

### 4.2 Sensors

The production of ionic oxygen species on the surface of the ZnO QDs is more favorable as compared to that on nanoparticles since there are more conduction band electrons on the surface of the QDs, resulting in the formation of a thick electron depletion layer. Thus, the electron requires a large amount of energy to migrate due to creation of a high potential barrier. On the adsorption of gas molecules, there is a drop in the potential barrier and resistance. These properties are favorable for the fabrication of gas-sensing devices. Ratan *et al.*^148^ have fabricated an interdigitated metal–semiconductor–metal (MSM)-based hydrogen gas sensor. This sensor shows high selectivity towards H_2_ with respect to sulfur dioxide, ammonia, and organic vapors such as methanol, chlorobenzene, acetone, and chloroform. Such gas sensing and selectivity towards a specific gas was also observed by Shouli Bai and his group.^149^ They fabricated ZnO QD-based sensors, which show a sensitive response for NO_2_ in comparison to CO and CH_4_. Zhang *et al.*^150^ synthesized water-soluble Cd-doped ZnO QDs and utilized the fluorescence-linked immunoassay (FLISA) method to inspect the level of bisphenol A (BPA) in water. Eu-Doped ZnO QDs are very helpful in the detection of *Bacillus anthracis* spores, which causes highly contagious diseases in human beings.^151^

The sensing mechanism of the QD-based sensors is quite different as compared to nanoparticle-based sensors. The change in the electrical conductivity is the basis of the sensing property of the nanoparticles.^152–154^ The red-emitting Eu(iii) ion acts as a signal reporting unit *via* chelation with calcium dipicolinate (CaDPA), which is the biomarker of *Bacillus anthracis* spores.

### 4.3 Biosensors

The production of ionic oxygen species on the surface of the ZnO QDs is not the sole accountable mechanism for the sensing properties of the ZnO QDs. Chemical detection, bio-imaging, bioanalysis, and cold-illumination are the fields that are under the grip of chemiluminescence (CL) due to its unique light emission process and high luminescence efficiency. In the present era, various inorganic nanoparticles such as Au, Ag, and CdTe have shown eye-catching special CL properties, which have attracted considerable attention of the researchers worldwide. However, their practical applications have been restricted by the relatively low quantum yield as compared to other organic materials. ZnO is emerging as a potential candidate for bio-imaging because of its excellent photoluminescence efficiency. Thus, functionalizing ZnO QDs with different molecules has unlocked the door not only for target-specific drug delivery but also for the detection of some specific enzymes and organic molecules, which are undetectable by bare ZnO QDs. J. P. Stelmaszyk^155^ and his team employed the fluorescence property of ZnO QDs for the detection of proteinase 3 (PR3) by functionalizing it with a peptide probe (Tyr-Tyr-Abu-Asn-Thr-Pro-OH) and an organic quencher (BHQ). This peptide probe significantly decreases the detection limit from 43 pmol to 1.3 pmol. This will be beneficial in the detection of diseases related to neutrophil activation and the over-expression of PR3. Due to the accumulative near-field effect of ZnO QDs, the enhanced fluorescence of *N*-acetyl-β-d-glucosaminidase (NAGase) is useful for the detection of *Streptococcus dysgalactia*, which is responsible for the prominent inflammatory disease, bovine mastitis (BM), in milk-producing animals.^41^

**Fig. 22:**
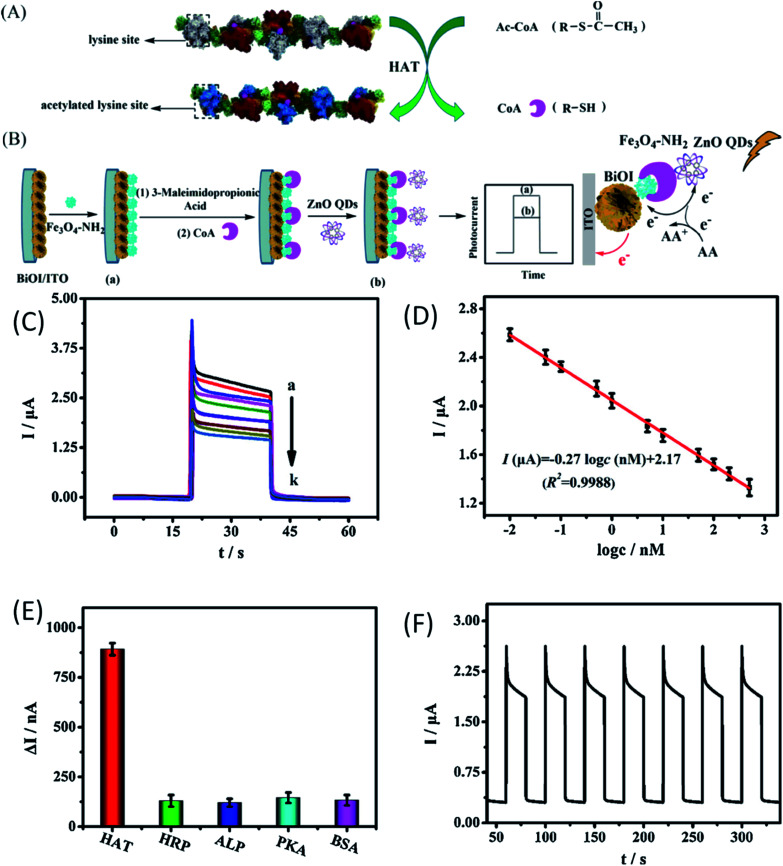
(A) Mechanism for the acetylation of the short peptide catalyzed by HAT, (B) schematic diagram of the construction of the PEC biosensor for detecting HAT, (C) the photo-electrochemical response of the biosensor with different concentrations of HAT. (D) Calibration curve of the biosensor for HAT detection. (E) The histogram for the photocurrent changes of the biosensor fabricated with different targets. The concentration of different targets was 100 nM. (F) Time-based photocurrent response of the biosensor toward 100 nM HAT.^156^ Adapted with permission from ref. 156. Copyright (2020) Elsevier.

Chen *et al.*^156^ have used QDs as a biosensor for the detection of histone acetylation using acetyl coenzyme A (Ac-CoA) as the target molecule. This is an indirect method for the detection of histone acetyltransferase (HAT). The schematic diagram for the detection mechanism is given in Fig. 22a and b. In this process, CoA is produced as a by-product of acetylation of Ac-CoA, which can be easily detected by the photo-electrochemical biosensor due to the presence of phosphate and thiol groups in its structure. From Fig. 22c, we can see that with the increase in the HAT concentration, the photocurrent also increases gradually. Fig. 22d shows a linear relation between the logarithmic concentration of HAT and the photocurrent intensity. From its wide range of linear relation, we can predict its potential for the detection of HAT. It shows high selectivity towards the HAT molecule in comparison to other molecules (as shown in Fig. 22e). It has relatively similar cycles for detection (as shown in Fig. 22f), having nearly 0.92% calculated relative standard deviation (RSD). Suppressing the working of electroluminescence reagents is emerging as an effective tool for the detection of biomolecules.^157^ β-Cyclodextrin (β-CD)-capped ZnO QDs decorated with pyridoxal 5′-phosphate (PLP) and pyridoxal (Py) is useful for the detection of histamine.^158^

### 4.4 Bio-imaging

**Fig. 23:**
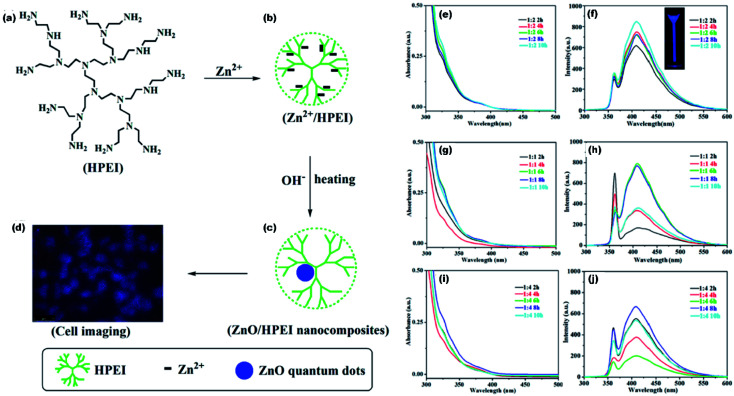
(a–d) Illustration of the ZnO/HPEI nanocomposite and the fluorescent cell imaging, UV-Vis and PL spectra of ZnO QDs at [Zn^2+^/OH^−^] molar ratio of (e and f) 1 : 2, (g and h) 1 : 1, and (i and j) 1 : 4.^124^ Adapted with permission from ref. 124. Copyright (2020) Elsevier.

High fluorescence, chemi-luminescence, and non-toxicity of ZnO QDs is helpful in bio-imaging, when combined with some suitable organic molecules. In the present era, various inorganic nanoparticles such as Au, Ag, and CdTe have shown special CL properties that have attracted considerable attention of researchers world-wide. However, their practical applications have been restricted by the relatively low quantum yield as compared to other organic materials. ZnO is emerging as a potential candidate for bio-imaging because of its excellent photoluminescence efficiency. Lei and his group^124^ prepared the ZnO QD-HPEI composite using the reflux method. The ethanolic solution of ZnO/HPEI nanocomposite, on characterizing with fluorescence microscopy, gives blue emission (shown in Fig. 23a–d). The UV-visible and photoluminescence spectra of ZnO/HPEI are shown in Fig. 23e–j. These spectra confirm that heating and change in the concentration do not affect the UV-visible and photoluminescence results.

**Fig. 24:**
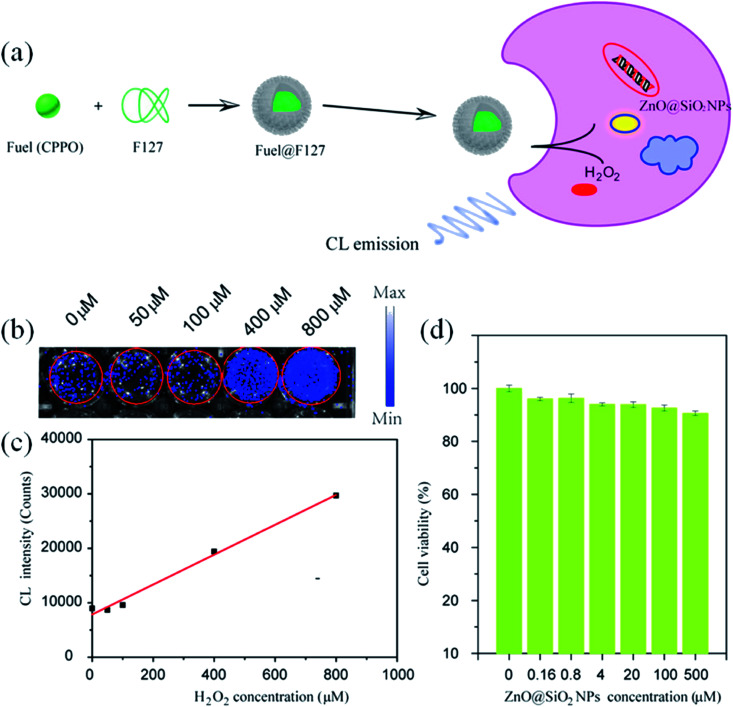
(a) Schematic illustration of ZnO NPs@SiO_2_-based CL bio-imaging. (b and c) CL images (b) and intensities (c) of the HeLa cells cultured with ZnO@SiO_2_ NPs for 6 h and then added into the mixture of CPPO at different concentrations of H_2_O_2_. (d) Cell viability of the HeLa cells after 24 h incubation in different concentrations of the ZnO@SiO_2_ NPs.^123^ Adapted with permission from ref. 123. Copyright (2020) Elsevier.

Similarly, Liu *et al.*^123^ have used ZnO QDs for the bio-imaging of HeLa cells. They synthesized the SiO_2_-coated ZnO QDs. For chemo-luminescence bio-imaging, ZnO/SiO_2_ was dissolved in ultrapure water and then it was used for staining the culture-grown HeLa cells. These samples were then added to CPPO, which was then cross-linked with F127 for improving the hydrophobicity of CPPO. After adding H_2_O_2_, this sample was ready for imaging. This whole process is shown in Fig. 24a.

With increasing H_2_O_2_ concentration, the intensity of imaging was also found to be increase (shown in Fig. 24b and c), which indicates a direct relation between H_2_O_2_ and the imaging intensity. However, the cell viability for different concentrations of ZnO/SiO_2_ after 24 h of incubation remains nearly constant (shown in Fig. 24d).

### 4.5 Optoelectronics

Beside gas sensing and bio-imaging, ZnO QDs can also be utilized for photo-sensors and photodetectors. Buddha Deka Boruah and Abha Misra^159^ fabricated a graphene-based ZnO QD photodetector with response and recovery time of nearly 0.29 seconds. Similarly, Gong *et al.*^91^ also fabricated a ZnO QD-based photodetector using graphene. They printed QDs directly on the graphene field effect transistor (GFET) channel. This utilizes the high mobility of graphene and the high electron density of ZnO QDs for the better performance of the device. The photoresponse of the ZnO QD-based photodetectors can also be increased with the aid of localized surface plasmon resonance (LSPR).^160–162^ In this technique, novel metal nanoparticles are fabricated over the sensing material. In such a case, a near-surface electric field is formed due to the collective oscillation of electrons on the surface nanoparticles, which ultimately increases the absorption of light. Liu *et al.*^160^ fabricated the ZnO QD thin film decorated with Au antennas. This type of photodetector has shown nearly 9.1 and 4.9 times increase in the photo-responsivity and normalized detectivity, respectively. The QDs have also been found to be applicable for the fabrication of LED.^163^ The excitation wavelengths of the UV range helps in developing ZnO-based visible light emitting QLEDs. The defect-related emissions of green, yellow, and orange-red from the ZnO QDs can be completely restricted by reducing the oxygen vacancies. The hybridization of the Zn interstitials with the anti-bonding O-states of graphene oxide (GO) QDs thus help in reducing these emissions and increasing the violet-blue emission.

**Fig. 25:**
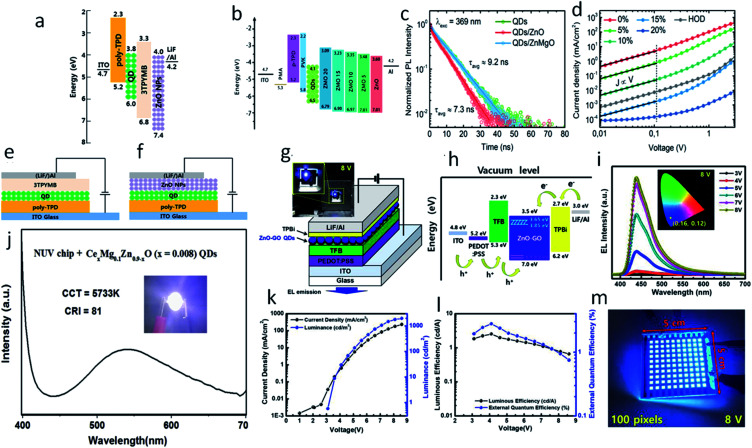
(a) Energy level of QLED.^164^ Adapted with permission from ref. 164. Copyright (2014) American Scientific. (b) Energy level of Mg-doped ZnO-based QLED, (c) TRPL spectra of Mg-doped ZnO and the QDs interface, (d) *J*–*V* curve of the Mg-doped ZnO device.^165^ Adapted with permission from ref. 165. Copyright (2020) Elsevier. The device structure of (e) 3TPYMB and (f) ZnO QDs.^164^ Adapted with permission from ref. 164. Copyright (2014) American Scientific. (g) ZnO–GO based QLED, (h) energy level diagram, (i) EL spectra of the ZnO–GO-based device.^16^ Adapted with permission from ref. 16. Copyright (2020) American Chemical Society. (j) EL spectra of Ce- and Mg-doped ZnO QLEDs.^115^ Adapted with permission from ref. 115. Copyright (2020) Elsevier. (k) Current density and luminance *vs.* voltage. (l) Luminous efficacy and external quantum efficiency (EQE) *vs.* voltage and (m) 100 pixel display of the ZnO–GO-based QLED.^16^ Adapted with permission from ref. 16. Copyright (2020) American Chemical Society.

The electron transport layer (ETL) plays a major role in any optoelectronic device. It should be capable of quickly transporting electrons so as to avoid charge recombination. Having high electron mobility, ZnO plays this role very effectively in most optoelectronic devices. Fig. 25g and h shows the schematic diagram of a ZnO–GO-based QLED device and the energy level diagram, respectively. Fig. 25i shows the blue electroluminescence (EL) of the device. Fig. 25k shows an increase in the luminance and the current density with voltage. Fig. 25l shows the nearly constant luminous efficiency and the quantum efficiency with increasing voltage. Fig. 25m shows the practical 100 pixels 8 V display of the blue QLED. It has been found that the ETL (electron transport layer) made of ZnO QDs has more current efficiency in comparison to organic ETL. The energy level diagram of the ZnO QD-ETL based device has been shown in Fig. 25a, which shows that the electron can be easily transported through the ETL to the Al cathode. It also helps in blocking the hole movements. Fig. 25e and f shows the device structure of the QLED-based on organic ETL and ZnO ETL. It was due to better charge balance by the ZnO QDs.^164^ The efficiency can be further increased with the help of doped ZnO QD-ETL.^165^ Doping in the ETL lifts the conduction band minima and reduces the electron mobility. The energy level diagram (Fig. 25b) shows a shift in the conduction band minima with the doping concentration of Mg. The TRPL spectra of the device (shown in Fig. 25c) shows a reduction in exciton quenching at the interface. This is very essential for improving the device performance. Fig. 25d shows a reduction in the current density with increasing Mg concentration. This is helpful in increasing the charge balance. Doping also results in an increase in the external quantum efficiency. Shi *et al.*^115^ fabricated Mg- and Ce-doped ZnO QDs LED for white light emission. The EL spectrum of the device is shown in Fig. 25e and the device image is shown in the inset. At 3 V potential and 200 mA driven current, this LED-correlated color temperature (CCT) and the color rendering index (CRI) were 5733 K and 81, respectively. For any optoelectronic device, ETL is an important part.^166^ Because of its low fabrication temperature and high electron mobility in comparison to TiO_2_,^167^ ZnO is one of the best candidates for ETL. Even in perovskite-based devices, ZnO is preferred as the ETL. In perovskite-based solar cells, ZnO increases the *V*_oc_, which results in an increase in the fill factor and efficiency.^167^ The high electron mobility of ZnO is useful in high electron extraction. It improves the band alignment with inorganic perovskite materials, improves the device stability, and reduces interfacial non-radiative recombination.^168^ The introduction of ZnO in inorganic perovskite films results in more compactly and uniformly distributed crystalline grains as it is evident that perfectly oriented grains considerably increase the power conversion efficiency.^169^ The ETL of ZnO in this case also enhances the transport of photo-generated carriers from the perovskite film to the electrodes, which improves the rise and fall time of perovskite-based photodetectors.^170^

Transparent nanocomposites made from ZnO QDs, SiO_2_, and epoxy^171^ for encapsulating LED^172^ would be highly beneficial in the near future. Besides encapsulating LED, nanocomposites are also applicable in other fields. Li *et al.*^173^ prepared ZnO QD-based polymer composite using poly(methyl methacrylate) (PMMA) as the polymer for UV-shielding^87^ material. This material can be used in a number of applications such as contact lenses, UV-shielding windows, or glasses. It was also found that the ZnO QDs can initiate photo-degradation of the host polymer matrix, which can be applied for recording materials. Georgia G. Goourey^81^ and his team studied the effect of ZnO nanoparticles with size ranging from 5 to 30 nm on acrylate photopolymers. They found partial quenching of the yellow green fluorescence with the incorporation of the QDs, whereas with the increase in the size to 30 nm, photo-degradation decreases drastically. ZnO QDs were also found to degrade organic compounds, which are poisonous to human health. Fakhri *et al.*^82^ prepared the ZnO QD/CuO composite for the degradation of the Tetanus toxin. They found 75% degradation under UV irradiation.

### 4.6 Photocatalysis

**Fig. 26:**
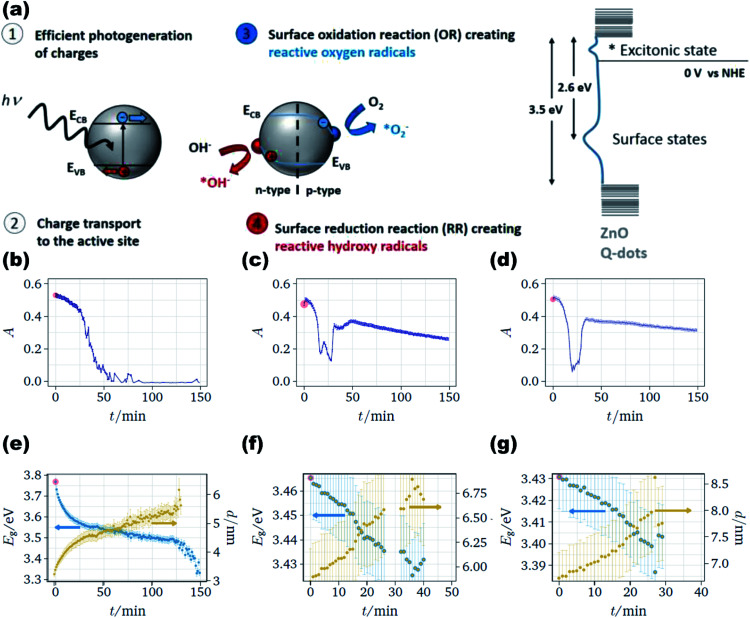
(a) Schematic diagram for charge generation on the surface of the ZnO QDs and the energy level diagram, absorption spectra of the dye for the decolorization process with initial diameter (b) 3.2 nm, (c) 5.8 nm, (d) 6.7 nm, and band gap and size of the growing ZnO QDs for particles with starting diameters of (e) 3.2 nm, (f) 5.8 nm, (g) 6.7 nm.^24^ Adapted with permission from ref. 24. Copyright (2020) American Chemical Society.

The smaller size of the ZnO QDs is beneficial over nanoparticles in the field of photocatalysis as the interplay between the accessible surface area and the ability to absorb incident photons results in higher quantum efficiency of photocatalysis. Researchers are working on a slow-growing process, in which the particles can interact with their surrounding particles or their own particles and grow at the later stages of the process into a larger particle or aggregate. Particles developed in such a way could be an effective tool for the effective removal of emerging water pollutants such as pesticides, herbicides, pharmaceuticals, and fragrances. Some of the covalent bonds can be easily broken down by UV light but some of them need a photocatalyst. Thus, here begins the role of ZnO QDs, which degrades the chemical bond of some complex organic molecules *via* the production of reactive oxygen species such as superoxide radicals O_2_˙^−^, singlet oxygen ^1^O_2_, hydroperoxyl radicals HO_2_˙, hydroxyl radicals ˙OH, and hydrogen peroxide H_2_O_2_, which are generated at the metal oxide surface upon UV irradiation in aqueous solutions. The rate of charge generation in the ZnO QDs is much faster than that in other indirect semiconductors. In the presence of water molecules, the photogenerated holes generates the hydroxyl radical (˙OH) (shown in Fig. 26a). The photocatalytic degradation of the dye is faster in the presence of QDs having a smaller size. As the size of the QDs, increases the rate of degradation decreases but the kinetics are found to show an increase at the initial stage. The kinetics then remain constant for whole the process (shown in Fig. 26b–d). The size of the QDs increases with time and its energy band gap decreases with time (shown in Fig. 26e–g). Taha Ahmed and Tomas Edvinsson^24^ studied the photocatalytic application of ZnO QDs in colloidal suspensions, which shows a higher photocatalytic degradation of organic dyes for QDs between 3 and 6 nm.

### 4.7 Anti-microbial

Bacterial infection is a perpetual health problem, leading to complicated wound healing problems. Numerous efforts have been made by a number of researchers worldwide to overcome the contagious problem. Materials such as metal oxides, silver particles, and hydrogels are some of the antibacterial materials made by researchers. Recently, it has been found that the antibacterial property of metal oxide materials is more effective in comparison to other antibacterial materials. Among them, ZnO has been found to be the most promising because of its availability, biocompatibility, low-cost, negligible toxicity towards human cells, as well as antifungal and antibacterial properties. Liang *et al.*^43^ synthesized graphene oxide-modified ZnO QDs that possess excellent killing effect for *Staphylococcus aureus* (Gram-positive) and *Escherichia coli* (Gram-negative). The size of the ZnO QDs also affects the antimicrobial activity. As the size of the QDs decreases, the density of the charged carrier defects also increases on the surface of the QDs, which generates more and more ROS. These ROS are responsible for the killing process.

**Fig. 27:**
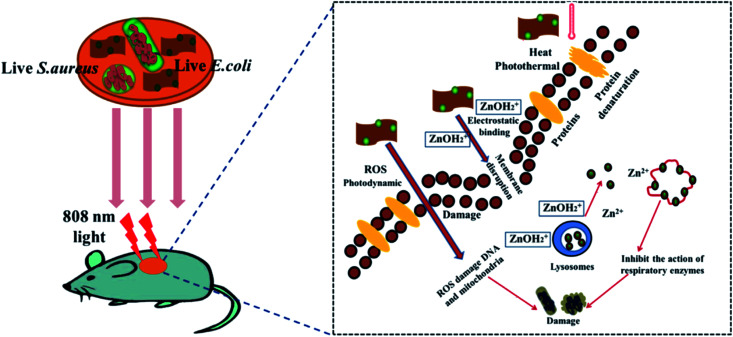
Schematic diagram for the killing mechanism of *Staphylococcus aureus* and *Escherichia coli* bacteria.^43^ Adapted with permission from ref. 43. Copyright (2019) Elsevier.

From Fig. 27, we can see the whole killing mechanism. In detail, the anionic membrane is first attacked by the cationic ZnO QDs composite, which destroys the cell membrane of the bacteria. When the composite attacks the membrane, it releases Zn^2+^ ions due to the lower pH level at that region. The absorption of Zn^2+^ on the surface of the membrane not only inhibits the respiratory action of the enzymes but also produces reactive oxygen species (ROS). ROS damages the membrane, DNA, and mitochondria. GO sheets produce hyperthermia, which prevents infections in the wound. Abinit Saha and his group^174^ found that QDs having particle size in the range of 3–5 nm have the maximum ability to destabilize the CRP (Cyclic AMP Receptor Protein) structure of the *E. coli* bacteria.

### 4.8 Cancer treatment

**Fig. 28:**
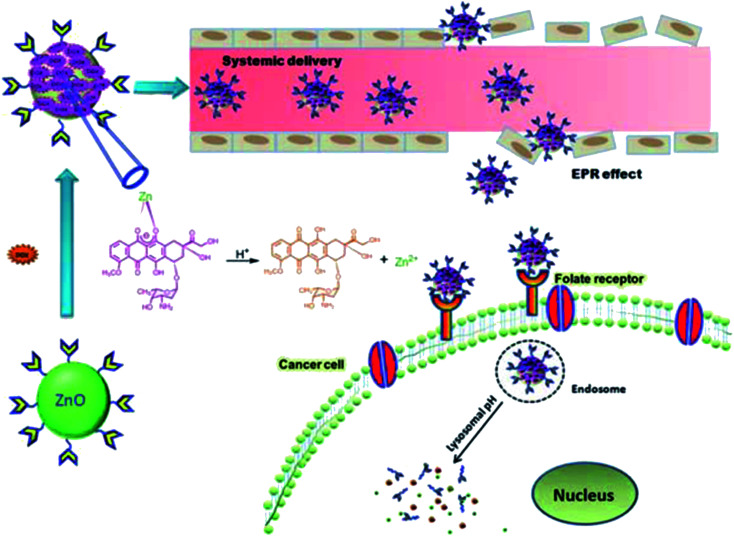
Schematic illustration of the synthesis and working process of folic acid-conjugated ZnO QDs.^42^ Adapted with permission from ref. 42. Copyright (2011) Royal Society of Chemistry.

ROS generation gives power to ZnO QDs so that it can also kill the cancer cells.^175^ For the efficacious operation of anticancer drugs, it is essential to deliver it at the tumor-specific region. The difference in the pH level of the extracellular and intracellular environment of the tumor tissues is very useful for targeted drug delivery. Many researchers have applied the pH responsive property of ZnO QDs for the targeted drug delivery of anticancer medicines. Arivazhagan *et al.*^80^ utilized the pH difference for target-specific drug delivery. They found that the microenvironment of MCF-7 and metastatic MDA-MB-231 human breast cancer tumor cells is more acidic than that of other normal healthy cells. For such a case, ZnO QDs are highly cytotoxic as compared to other nanostructures of ZnO. Faheem Muhammad and his group^42^ obtained this property by conjugating folic acid on to the surface of the ZnO QDs. Doxorubicin (DOX) and ZnO QDs were simultaneously found to be more efficacious than alone. Fig. 28 shows the loading of DOX on folic acid-capped ZnO QDs. Next, we can see that these particles are preferentially accepted at the cell membrane of the cancer cell by the folate receptors. Here, due to the appropriate pH conditions, the DOX drug is released, which results in the killing of the cancer cell.

Lastly, we can say that ZnO can help in cancer treatment not only by its killing ability but also by acting as a drug carrier. It could play a major role in overcoming the challenges such as recurrence of disease, drug resistance, and side-effect of the drug. Drug resistance is found with many known anti-cancer drugs currently in use. Recurrence poses a challenge that is also related to these known drugs. ZnO will be very helpful in overcoming these challenges. Its drug delivery mechanism will improve the selectivity of the drug towards cancer cells, which finally increases its effectiveness. It will also reduce the possibility of drug side-effects as it will only act at the specific site.

### 4.9 Li-Ion batteries

Beside these widely-accepted applications, some miscellaneous applications of ZnO QDs also exist. These are not as popular as the above applications, probably because of their low feasibility. But research is still going on in these fields with the hope of developing an efficient tool that may remove the barrier and make it feasible. The use of ZnO QDs for the storage of lithium ions in lithium-based batteries is one such application. The Li-ion storage properties of different ZnO QD-based composite materials are shown in Table 2. It is found that the anode material made up of ZnO has a large theoretical capacity of nearly 987 mA h g^−1^. However, a major challenge in the use of ZnO as the anode material is the huge volume change and low electron conductivity. To overcome this problem, an effective method is to use graphene or its derivatives in ZnO-based anode materials. RGO-based ZnO QD composites have found to show high electrochemical performance for Li-ion storage, with a high reversible capacity of 800 mA h g^−1^ even after 200 cycles and stable reversible capacity of 668 mA h g^−1^ at a high current density of 1000 mA g^−1^ even after 700 cycles. This is because of the conservation of the electrode structure, which allows the smooth flow of the Li-ion during charging and discharging. The anchoring of ZnO QDs on the supporting framework of RGO provides such an excellent stabilization.^117^

**Table 2:** Comparison of the lithium-ion storage properties of various ZnO QD-based composites

S. no.	Sample name	Reversible capacity (mA h g^−1^)	Cycle life	Capacity retention (%)	Current rate (mA g^−1^)	Ref.
1	Amorphous ZnOQDs/MPCBs	930	85	90	100	176
		840	280	93.1	200	
		510	400	94	1000	
2	ZnO-VAGNs	809	100	93	80	177
		450	250	87.7	350	
3	ZnO-QDs@CMS	1015	80	—	50	178
		565	350	94.3	1000	
4	ZnO@ZnO QDs/C NRA	699	100	100	500	179
5	ZnO QDs@porous carbon	1150	50	67.6	75	180
6	ALD ZnO/G	∼540	100		100	181
7	ZnO/RGO	∼800	200	104	200	117
		668	700	87	1000	

**Fig. 29:**
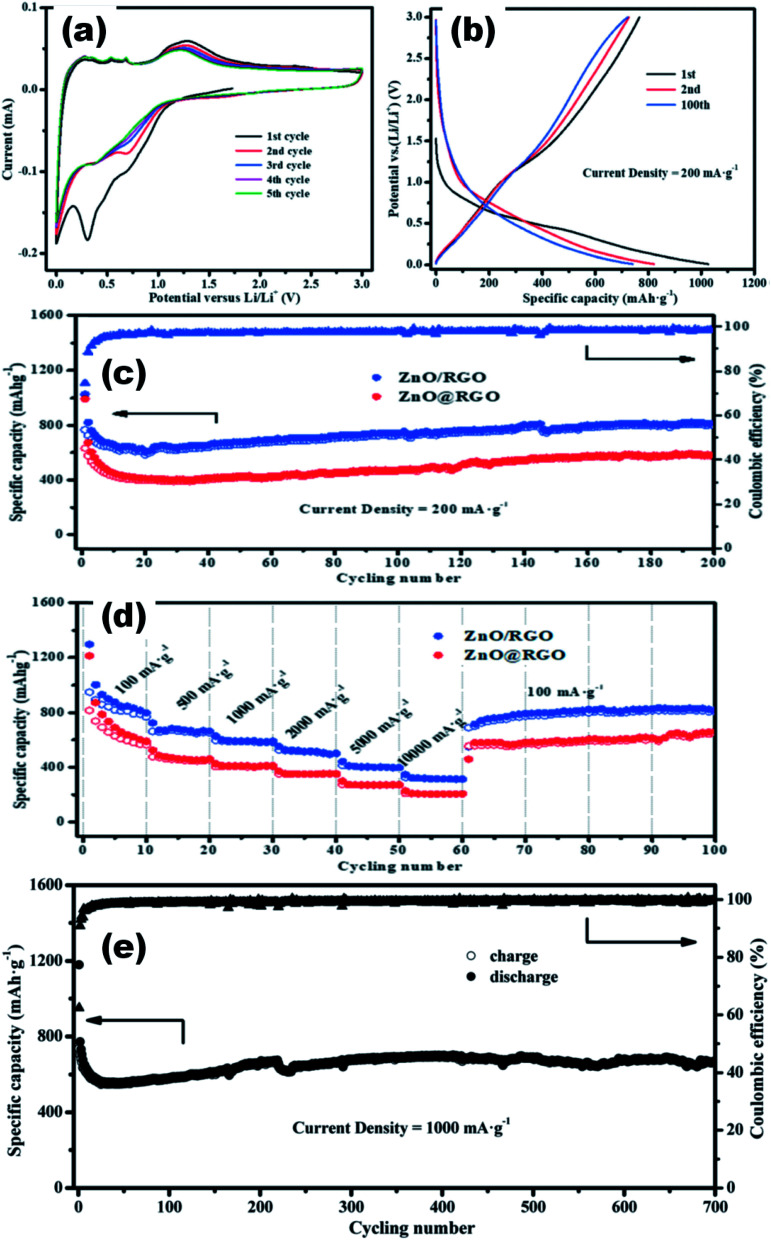
(a) CV curve for 5 initial cycles, (b) charge–discharge curve, (c) cycling performance, (d) rate performance, and (e) cycling performance at 1000 mA g^−1^ for the ZnO/RGO composite electrode.^117^ Adapted with permission from ref. 117. Copyright (2020) Royal Society of Chemistry.

Initial cathodic scanning shows a peak at 0.31 V, which is attributed to the reduction of Zn^2+^ to Zn metal and the generation of a solid electrolyte interphase. The peaks at 0.69 and 0.37 V are due to the reduction of ZnO to Zn and the formation of the Li_*x*_Zn alloy with Li^+^, respectively. The de-alloying of Li_*x*_Zn is clearly visible from the four peaks of the anodic scanning curve at 0.27, 0.36, 0.55, and 0.68 V. A large peak at 1.3 V in the anodic scanning curve is due to the formation of ZnO in the reaction of Zn and Li_2_O (shown in Fig. 29a). From Fig. 29b, we can see that the first charging and discharging curve shows a voltage at 1.3 V and a large plateau at 0.3 V. The initial discharge and charge capacity were 1027 and 766 mA h g^−1^, respectively, which gives an initial coulombic efficiency of 74.6%. The cyclic performance of the ZnO/RGO composite electrode and ZnO@RGO at a current density of 200 mA g^−1^ gives 766 mA h g^−1^ initial reversible capacity, which drops to 605 after 20 cycles (shown in Fig. 29c). After 30 cycles, the coulombic efficiency stabilizes above 97% and after 200 cycles, the reversible capacity reaches 800 mA h g^−1^. Fig. 29d shows the average reversible capacities of 840, 670, 590, 515, 400, and 315 mA h g^−1^ at 0.1, 0.5, 1.0, 2.0, 5.0, and 10.0 A g^−1^, respectively, for the ZnO/RGO composite electrode. Small ZnO size and the large and thin supporting structure of RGO are responsible for the excellent rate capability of the ZnO/RGO electrode. The cyclic performance of the ZnO/RGO electrode at a higher current density of 1000 mA g^−1^ is shown in Fig. 29e.

In summary, the main applications of doped and undoped ZnO QDs are found to in antibacterial, antifungal, and anticancer medicines. Other applications are the sensing and detection of toxic materials. Quantum confinement enhances the properties of the ZnO QDs, which opens the door for noble applications. pH-responsive drug delivery will be game changing in the treatment of cancer. The photocatalytic activity will revolutionize its application in different chemical industries and environment-related challenges. Graphene and polymer-based composites can widen the application field of ZnO QDs. Graphene and reduced graphene-based ZnO composites are better candidates for storage applications in the future.

## 5. Conclusion

In recent years, a lot of work has been done by researchers worldwide in the field of ZnO QDs. The quantum confinement effect predominantly emerges nearly in all the properties of QDs. It has manipulated the whole character, as we have seen in the case of ZnO nanoparticles. The sol–gel method is the most suitable synthetic method for the preparation of ZnO QDs. With the variation in the used solvent and the precipitating agent, different physical properties can be altered. The important factor that always affects the dot formation is the pH of the solution, which should not be below 7 and should be higher than 10 as a pH less than 7 does not favor the process to start and a pH more than 10 pH results in the agglomeration of the particles. The essence of the whole manuscript is given in the following points.

(1) With a reduction in the particle size below 8 nm, strong quantum confinement effect comes into play. Due to exciton–phonon interaction, the ground state energy and oscillator strength reduces with the particle size of the QDs. Polaronic self-energy corrections of the exciton vanishes completely and the PB potential effectually transforms into a dynamically-screened Coulomb potential.

(2) Longitudinal optical (LO) and transverse optical (TO) modes of bulk ZnO splits into A_1_ and E_1_ symmetries in ZnO QDs. The phonon peak shift that arises in the ZnO QDs is related to three main factors: phonon localization by defect creation, confinement effect within the QD boundaries, and the laser-induced heating effect.

(3) The fluorescence intensity of the QDs increases with the doping of elements. But after a certain limit, it starts decreasing. The fluorescence intensity is quenched with the addition of any metal ion impurity or organic ligand. This property is useful for fluorescence probes. The surface charge density also increases in the QDs.

(4) The sol–gel method is the most popular as it is a relatively more efficient, simple, and inexpensive method over others. With the help of this method, QDs stable in water and other alcohols (mostly ethanol) can be prepared. Composites made *via* SiO_2_, graphene, and PMMA are able to increase the stability of the QDs. Radio frequency-based synthesis is emerging as a new technique for QD synthesis.

(5) Most of the doping elements are found on the surface of the QDs. With the help of the core/shell (ICS) procedure, we can inject doping elements inside the core of the ZnO QDs. The doping of rare earth elements is difficult as compared to transition metal doping. Rare earth elements are useful for optical properties and transition metal doping induces magnetism in the QDs.

(6) Rare earth doping is mostly favorable for the variation in the photoluminescence-related properties, whereas transition metal doping mostly induces magnetic behavior in the QDs. Both types of doping introduces photocatalytic character in the QDs by producing different vacancies.

(7) The detection of metal ion impurities (Fe^2+^, Cr^6+^, Hg^2+^, Cl^−^, *etc.*) distinguishes it from nanoparticles. The interaction of graphene and QDs is helpful in optoelectronic device fabrication. The enhancement in ROS generation increases its anti-bacterial and anti-microbial activity. ROS generation also increases the cytotoxicity of the QDs. An effective drug delivery mechanism sharpens the QDs' cytotoxicity. Li-Ion storage can be achieved by hanging QDs in the framework of graphene or graphene-derived components.

(8) Having such a wide field of application, ZnO QDs have a very bright future. They can be used as an impurity-detecting tool. They can be used for the production of low-cost gas sensors. They can replace TiO_2_ from the ETL in many optoelectronic devices. Apart from their commercial use in cosmetics, they can also be used in anti-microbial and anti-bacterial ointments. Their use in the treatment of cancer will definitely increase the effectiveness of the drug and will decrease the side-effects of the drug at the same time.

## Conflicts of interest

There are no conflicts to declare.

## Supplementary Material
